# EBV-Positive Lymphoproliferations of B- T- and NK-Cell Derivation in Non-Immunocompromised Hosts

**DOI:** 10.3390/pathogens7010028

**Published:** 2018-03-07

**Authors:** Stefan D. Dojcinov, Falko Fend, Leticia Quintanilla-Martinez

**Affiliations:** 1 Department of Cellular Pathology, University Hospital of Wales, Cardiff CF14 4XW, UK; Dojcinov@cardiff.ac.uk; 2 Institute of Pathology and Neuropathology and Comprehensive Cancer Center Tübingen, University Hospital Tübingen, Eberhard-Karls-University, 72076 Tübingen, Germany; falko.fend@med.uni-tuebingen.de

**Keywords:** Epstein-Barr virus, lymphoproliferations, lymphoma, epidemiology, pathogenesis, morphology, clinical features

## Abstract

The contribution of Epstein-Barr virus (EBV) to the development of specific types of benign lymphoproliferations and malignant lymphomas has been extensively studied since the discovery of the virus over the last 50 years. The importance and better understanding of the EBV-associated lymphoproliferative disorders (LPD) of B, T or natural killer (NK) cell type has resulted in the recognition of new entities like EBV+ mucocutaneous ulcer or the addition of chronic active EBV (CAEBV) infection in the revised 2016 World Health Organization (WHO) lymphoma classification. In this article, we review the definitions, morphology, pathogenesis, and evolving concepts of the various EBV-associated disorders including EBV+ diffuse large B-cell lymphoma, not otherwise specified (DLBCL, NOS), EBV+ mucocutaneous ulcer, DLBCL associated with chronic inflammation, fibrin-associated DLBCL, lymphomatoid granulomatosis, the EBV+ T and NK-cell LPD of childhood, aggressive NK leukaemia, extranodal NK/T-cell lymphoma, nasal type, and the new provisional entity of primary EBV+ nodal T- or NK-cell lymphoma. The current knowledge regarding the pathogenesis of B-cell lymphomas that can be EBV-associated including Burkitt lymphoma, plasmablastic lymphoma and classic Hodgkin lymphoma will be also explored.

## 1. Introduction

Epstein-Barr virus (EBV) is a ubiquitous gamma herpes virus with tropism for B cells. EBV is the most common persistent virus infection in humans with approximately 95% of the world’s population showing an asymptomatic life-long carrier status [1]. This sustained life-long latent infection is the result of the unique interaction of EBV with B cells, specifically memory B cells, which are the EBV reservoir in healthy individuals [2,3]. The disruption of this finely regulated balance between virus and host immune system can result in EBV-associated lymphoproliferations (LPD) of B, T and NK cell derivation.

Primary infection is usually asymptomatic and occurs early in life, and when symptomatic is usually a self-limited disease occurring in adolescents or young adults manifested as acute infectious mononucleosis (IM) [4]. IM is characterized by a polyclonal expansion of infected B-cells that triggers a cytotoxic T cell response resulting in a transient, antigen-driven oligoclonal expansion of CD8+ T-cells [5,6]. Both the quantity and quality of the CD8+ T cell response to EBV are critical to control the infection [7,8]. In order to escape T cell immunosurveillance, EBV downregulates antigen expression and establishes a stable reservoir of memory B cells, in which latent viral proteins are no longer expressed and EBV genome remains in episomal form [1,9,10,11]. There are three EBV latency patterns recognized [2,3]. Latency III involves the unrestricted expression of all nine latent genes including six EBV-encoded nuclear antigens (EBNA1, 2, 3A–C and LP) and three latent membrane proteins (LMP1, LMP2A and LMP2B). This latency program occurs only during acute EBV infection or in severely immunodeficient individuals. These viral proteins are highly immunogenic and trigger a strong cytotoxic T-cell reaction. Latency II is an intermediate pattern with expression of many proteins except for EBNA2. EBNA2 and LMP1 are the main transforming proteins of EBV. The expression of EBNA2 is important to demonstrate a latency type III whereas the expression of LMP1 in the absence of EBNA2 is used to confirm latency type II. Latency I is restricted to the expression of EBNA1, which is expressed in all virus-infected cells and is responsible for the maintenance and replication of the episomal EBV genome. The so-called latency 0 is present in resting memory B-cells that carry the viral genome but viral antigen expression is maximally suppressed. The transcription of non-polyadenylated RNAs EBER1 and EBER2 is a constant feature of all latent EBV infection patterns, and therefore, represents the gold standard for identification of EBV in tissue sections [12]. In vivo, in addition to B cells, EBV is capable of infecting T and natural killer (NK) cells, as well as some epithelial and mesenchymal cells. The infection of the former cell types may lead to several EBV related LPDs, with disease manifestations generally depending on the type of EBV infected cells and the state of host immunity [11]. In the assessment of EBV-associated LPDs, particularly in immunosuppressed states, serological investigation of EBV copy numbers provides valuable information regarding the extent of immunosuppression or burden of EBV positive lymphoproliferation. Interpretation of these results should be made in a multidisciplinary context. EBV-positive LPDs encompass disease entities with broad clinico-pathological spectrum. According to the infected cell type, the EBV-positive LPDs can be grouped in B and T/NK-cell categories (Table 1 and Table 2). The focus of this review is to describe the clinico-pathological features of the well-known B-, T- and NK-cell EBV-associated lymphomas and lymphoproliferations in non-immunocompromised hosts [13].

## 2. EBV Associated B-Cell Lymphoproliferative Disorders

EBV positive B-cell LPDs represent a wide and expanding clinico-pathological spectrum ranging from indolent, self-limiting and localized conditions to highly aggressive lymphomas. Particularly in the recent years there has been an increased understanding of indolent EBV positive B-cell LPDs, which have historically been regarded as aggressive neoplasms and managed as such (e.g., EBV positive mucocutaneous ulcer (EBV+ MCU) and fibrin associated diffuse large B-cell lymphoma FA-DLBCL). While EBV represents the common denominator, it is variably involved in aetiology and pathogenesis, and other factors, including underlying genetic alterations (such as *MYC* rearrangement), the inherent state of the host immune response or iatrogenic immunosuppression play important pathogenetic roles [13]. EBV positive B-cell LPDs affect all ages and are prevalent worldwide, but the incidences of different entities show wide geographical variation. Those in which EBV appears to be of crucial pathogenetic role are particularly prevalent in areas with high rates of early EBV infection such as parts of Africa, Asia or South America (e.g., endemic Burkitt lymphoma (BL) or EBV positive diffuse large B-cell lymphoma, NOS (EBV+ DLBCL)). Overall, the most common EBV associated B-cell LPD in the Western population (EBV+ DLBCL) represents approximately 3% of lymphomas but is much more prevalent (7–15%) in South America and Asia. In contrast, some are very uncommon in everyday practice (e.g., lymphomatoid granulomatosis (LyG) or FA-DLBCL) making them diagnostically and therapeutically problematic due to limited experience [13].

In this review, we will focus on the B-cell entities in which EBV is considered a defining diagnostic parameter, and where significant knowledge has recently been acquired, resulting in classification changes and better understanding of pathogenesis (Table 3). These include EBV+ DLBCL, diffuse large B-cell lymphoma associated with chronic inflammation (DLBCL-CI), EBV+ MCU, FA-DLBCL, and LyG. In addition, the entities in which EBV is detectable but does not represent the disease defining feature including plasmablastic lymphoma (PBL), BL and classic Hodgkin lymphoma (CHL) will be addressed. Those lymphomas in immunosuppressed patients, where EBV is considered a non-essential component of lymphomagenesis (e.g., the spectrum of post-transplant lymphoproliferative disorders (PTLD) or those associated with primary immunodeficiencies) are beyond the scope of this review. Infectious mononucleosis (IM) is briefly addressed as it frequently represents a significant diagnostic challenge.

### 2.1. Infectious Mononucleosis

Clinical descriptions of the syndrome corresponding to IM date to the early 19th century, but the term was for the first time used by Sprunt and Evans in 1920 in the Bulletin of Johns Hopkins Hospital, postulating infectious aetiology [14]. The link to EBV infection was described by Henle in 1968 after one of the laboratory staff at the Children’s Hospital in Philadelphia handling infected BL cultures developed IM. This study represented a foundation for serological diagnosis [15]. IM is an acute clinical manifestation of EBV infection characterised by reactive, self-limiting lymphoproliferation and an inflammatory syndrome. Whereas the majority of the immunocompetent population worldwide acquires lasting and effective immunity to EBV through asymptomatic infection, a minority presents with acute viral illness as a result of the viral lytic cycle. IM is most commonly seen in adolescents and rarely in children and adults, affecting both sexes equally [16]. The diagnosis is made on clinical and serological grounds by the detection of heterophilic antibodies to EBV (Monospot or Paul-Bunnell-Davidsohn test) or IgM antibodies against viral capsid antigen. However, a minority of patients undergoes a biopsy, the interpretation of which may be challenging and may led to an erroneous diagnosis of lymphoma. Hence, IM represents one of the pathologically most misdiagnosed reactive lymphoproliferations, with potentially severe clinical consequences [17].

#### 2.1.1. Clinical Features

The patients present with a febrile illness, a brief history of rapidly developing tonsillar enlargement, tender cervical lymphadenopathy and splenomegaly. Reactive lymphocytosis is seen. The disease is of short duration and self-limiting in majority of cases. Spontaneous splenic rupture occurring in 0.5% of cases is a potentially fatal complication.

#### 2.1.2. Morphology

**Figure 1:**
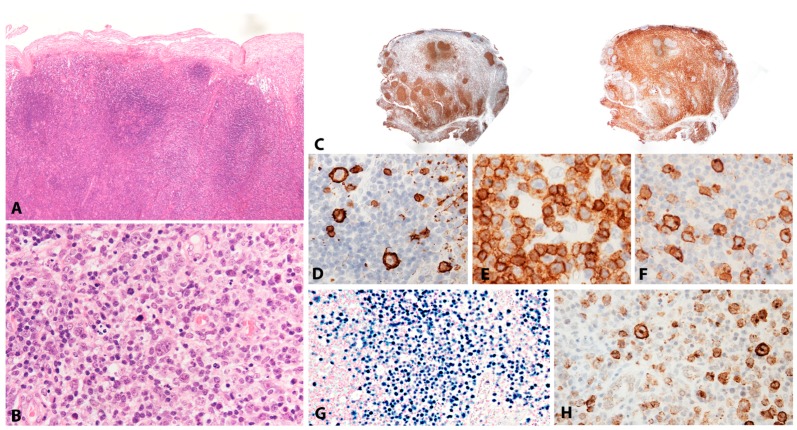
Infectious mononucleosis. (**A**) The lymph node shows retained architecture, hyperplastic lymphoid follicles and expanded paracortex (H&E, 100×). (**B**) The paracortex contains a polymorphous infiltrate of lymphocytes and plasma cells with numerous prominent immunoblasts, including some with Hodgkin-like features (H&E, 200×). (**C**) CD20 (left) and CD3 (right) highlight retention of lymph node architecture and separation of B-cell and T-cell compartments (magnification, 5×). (**D**) CD20 highlights prominent B-cell immunoblasts including those with Hodgkin-like features. (**E**) CD3 highlights abundant paracortical small lymphocytic T-cell infiltrate but also scattered large T-cell immunoblasts. (**F**) The immunoblasts and Hodgkin-like cells are CD30 positive. (**G**) There is abundant paracortical staining with EBER co-localising with the B-cells (*in-situ* hybridization, 100×). (**H**) The same cells are also positive for LMP1 (immunohistochemistry, D, F, H, 200×; E, 400×).

Lymphoid tissue from patients with IM (usually tonsils or cervical lymph nodes) shows a mixture of reactive patterns including follicular hyperplasia, perifollicular and sinusoidal monocytoid proliferation and paracortical hyperplasia (Figure 1A). The proportions of these reactive patterns are variable. In addition, wide, circumscribed areas of necrosis are often seen. The paracortex contains a polymorphous mixture of lymphocytes, plasma cells and particularly prominent immunoblasts which may form aggregates. Hodgkin and Reed-Sternberg (HRS)-like cell are common and may prompt a differential diagnosis with cHL or DLBCL in approximately 20% of cases (Figure 1B). Immunohistochemistry highlights retention and separation of T-cell and B-cell compartments, which is particularly useful in small needle core biopsies (Figure 1C). The hyperplastic lymphoid follicles show a reactive pattern of expression of markers including CD10, BCL6, BCL2, CD21, CD23, Ki67 and IgD, but occasionally small, BCL2 positive primary B-cell follicles compressed against the capsule by the expanded paracortex are seen. The paracortex is immunophenotypically polymorphous. A simple and useful diagnostic clue in difficult cases is presence of both T- and B-immunoblasts, which should make a diagnosis of lymphoma unlikely (Figure 1D,E). The B-immunoblasts, which may be particularly pleomorphic and with Hodgkin-like features, display a post-germinal centre phenotype (CD45+, CD20+, CD79a+, PAX5+, Oct2+, BOB1+, BCL6+/−, MUM1+). Most of the cells show expression of CD30 but lack of CD15 expression (Figure 1F). EBERs, EBNA2 and LMP1 show widespread positivity in the paracortex, including both the large immunoblasts and small B-cells, which may be important in excluding CHL, in which only the large HRS cells are positive (Figure 1G,H) [17].

#### 2.1.3. Pathogenesis and Molecular Findings

The patterns of EBV infection and establishment of lytic phase and latency have been extensively studied [7,18,19], and will be reviewed elsewhere in this series.

### 2.2. EBV Positive Diffuse Large B-Cell Lymphoma, Not Otherwise Specified

EBV+ DLBCL is a monoclonal EBV+ lymphoproliferation, first described in Japanese, European and North American patients over the age of 60 years. The patients presented with mostly extranodal, clinically highly aggressive disease and had no history of immunosuppression [20,21]. Initially referred to as “Senile EBV associated B-cell lymphoproliferative disorder” [22], it was included as a provisional entity in the 2008 WHO lymphoma classification as “EBV positive diffuse large B-cell lymphoma of the elderly” [23]. The key clinical diagnostic requirements included lack of known immunodeficiency, no prior history of lymphoma and patient age greater than 50 years. However, the age cut-off was rather arbitrary. After the original description, a similar spectrum of lymphomas was noted in much younger adults and in children [24,25,26]. As a result, in the 2016 WHO classification, the terminology was changed to “EBV positive diffuse large B-cell lymphoma, not otherwise specified”, thus removing the age denominator. Other well characterized EBV+ associated lymphomas like LyG, PBL, DLBCL-CI and EBV+ MCU, must be excluded [13]. EBV+ DLBCL is seen worldwide with a variable incidence between 2–15%, of all lymphomas. It is most prevalent in East Asia (13–15%), followed by Mexico (7%), and rare in Europe (2–3%) [20,27,28]. It is slightly more common in males (1.5:1) with a median age of 71, but some patients are as young as 4 years [20,21,24,26,29].

#### 2.2.1. Clinical Features

The elderly patients present with bulky extranodal disease (40–70%) or with significant local or generalised lymphadenopathy, accompanied by B-symptoms (40%). Almost any anatomical site can be involved, most often being lungs, upper aero-digestive and gastrointestinal tract. The affected elderly patients present with a high international prognostic index (IPI) and poor performance status. The younger patients have much less extranodal involvement (11%) and present mostly with lymphadenopathy [20,21,24,25,26,28,29]. Serum EBV DNA is identified in the majority of patients, the levels of which seem to correlate with the disease burden [30]. The survival of the elderly patients is generally poor, averaging 24 months. Many receive palliative or reduced intensity chemotherapy due to frailty. However, the younger patients (<45) fare much better, with approximately 90% long-term survival [24,31,32].

#### 2.2.2. Morphology

**Figure 2:**
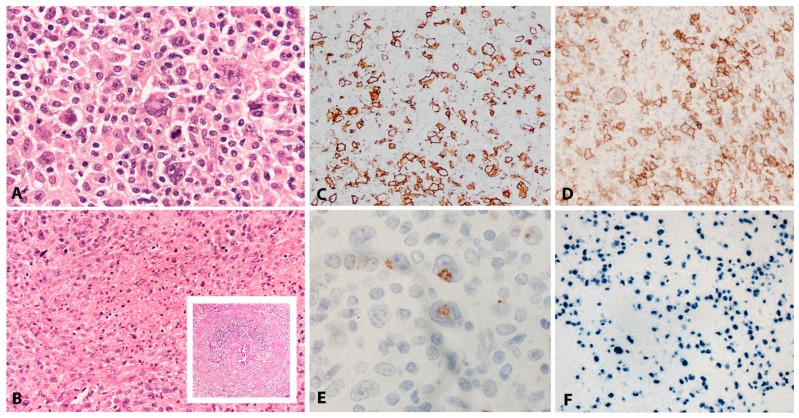
EBV positive diffuse large B-cell lymphoma, not otherwise specified. (**A**) Polymorphous infiltrate of HRS-like cells in a background of lymphocytes and histiocytes (H&E, 400×). (**B**) Wide areas of necrosis are common and invasion of vascular walls is seen (inset) (H&E, 100×). (**C**) The majority of tumour cells are positive for CD20, which highlights markedly variable size of the lesional cells. (**D**) Most of the tumour cells are positive for CD30. (**E**) Occasional cells co-expressing CD15 are seen. (immunohistochemistry, C, D, 200×, E, 400×) (**F**) There is widespread positivity for EBER, which highlights variability in the size of the nuclei. (*in-situ* hybridization, 200×).

EBV+ DLBCL displays a broad range of morphological features. Many show prominent CHL-like appearances with highly pleomorphic, large, HRS-like cells in an inflammatory background of histiocytes and lymphocytes, lacking eosinophils (Figure 2A). Some cases display a “T-cell/histiocyte rich B-cell lymphoma”-like morphology while others show more monomorphic appearance of conventional DLBCL. Cases with a polymorphous population of variably sized immunoblasts and plasma cells are also seen, and some display morphology reminiscent of PBL. Angioinvasion and geographic necrosis is a fairly constant feature (Figure 2B). The phenotype is mostly of post-germinal centre/”activated B-cell” lineage. There is variable expression of CD20, which is positive in at least 50% of the tumour cells, used as criteria for the differential diagnosis with CHL (Figure 2C) [33]. The cells are positive for CD19, CD79a, PAX5, OCT2, BOB1 and MUM1, with variable expression of BCL6 and CD45. CD10 is mostly negative [28,33,34]. There is usually positivity for CD30 and up to 68% of cases show co-expression of CD15, which makes the differential diagnosis with CHL difficult, especially in lymph nodes (Figure 2D,E) [21]. The majority of EBV+ DLBCL shows EBV latency II and rarely latency III. EBER is abundantly positive, showing variation in nuclear size (Figure 2F) [24,28].

#### 2.2.3. Pathogenesis and Molecular Findings

At the core of pathogenesis of EBV+ DLBCL are qualitative and quantitative changes in the immune competence and immune surveillance over EBV infection as a result of age. The complex spectrum of these changes results in immunosenescence, the naturally occurring process of decay of the immune system, which takes place with ageing and affects individuals variably. The underlying processes are multifactorial. There is diminished generation of lymphoid precursors, reduced thymic T-cell output, altered T-cell functionality, and “immune exhaustion” through lifelong antigenic exposures. As a result, the naïve CD8+ EBV specific T-cells pool is diminished and replaced by senescent and functionally inferior effector memory cells. At its most advanced stage, immunosenescence results in a hundred-fold reduction in T-cell antigenic repertoire [35,36,37]. Such changes generate an environment similar to iatrogenic immunosuppression, resulting in EBV latency III and EBV driven B-cell lymphoproliferation [29]. Recent EBV+ DLBCL cell culture profiling data indicates potential implication of EBNA3B mutational changes in the tumour cells resulting in lack of attraction of T-cell responses by tumour cells and more aggressive clinical behaviour [38]. No definite precursor lesions or identifiable risk factors have been established in the course of EBV+ DLBCL pathogenesis. However, elderly patients with presumed immunosenescence can develop a range of EBV positive, polyclonal hyperplasias at a median age of 67, 10 years younger than patients with EBV+ DLBCL. These include IM-like paracortical hyperplasia and follicular hyperplasia with EBV+ germinal centres. Recently, both have been found preceding or concomitant with EBV+ DLBCL [39,40,41]. It is yet unclear if these represent precursors to the development of EBV+ DLBCL or, more likely, an early reflection of an immunosuppressed state suggesting a role of immune response in controlling germinal centre transit of EBV infected cells [18]. In the vast majority of EBV+ DLBCL clonal IGH rearrangements are detected. In addition, as a result of senescent and reduced T cell repertoire, 60% of cases show “restricted” or oligoclonal T-cell receptor gene rearrangements by PCR [21]. By gene expression profiling EBV+ DLBCL shows activated B-cell phenotype with enhanced activity of the NF-κB and JAK/STAT pathways [34]. These lymphomas show a “host immune response” signature in which antiviral response genes, proinflammatory cytokines, and chemokines associated with the innate immune response are overexpressed, indicating the presence of a virus-induced inflammatory microenvironment. The genes associated with the B-cell receptor signalling pathway are downregulated and supplemented by EBV-mediated activation of NF-κB. EBV+ DLBCL harbours chromosomal gains at 1q23.2–23.3 and 9p24.1 co-localizing with key immunoregulatory genes encoding SLAMF1 and PDL2. Particularly the 9p24.1 gains result in upregulation of PDL2, immune evasion and, hence, poor prognosis. Interestingly, these genetic features are in many ways similar with those seen in EBV positive CHL [42,43,44,45]. The recent description of EBV+ DLBCL in young individuals highlights significant differences in the presentation and behaviour within this lymphoma type, potentially pointing to different pathogenetic mechanisms. The latency II in the younger population points to a lesser role of the host immune competence in pathogenesis and possible greater role of involvement of immune evasion [24].

### 2.3. EBV Positive Mucocutaneous Ulcer

EBV positive mucocutaneous ulcer is an ulcerating EBV+ B-cell lymphoproliferation affecting skin and mucosal surfaces, which pursues a self-limited indolent course. It was initially described in immunosenescent elderly patients but also in a range of immunosuppressed settings including HIV, PTLD, iatrogenic immunosuppression for autoimmune disorders and primary immunodeficiencies [46]. It shows CHL-like histological features, with EBV+ B-cells in a mixed inflammatory background with morphological and immunophenotypic similarities with EBV+ DLBCL, but with significantly different clinical characteristics and favourable outcomes. To distinguish it from the EBV+ DLBCL, it has now been included as a provisional entity in the 2016 WHO lymphoma classification [13]. This is a rare lymphoproliferation, which may be underestimated in its incidence because of its self-remitting character. Median age at presentation for immunosenescent elderly patients is 77 years, with a mild male preponderance. The iatrogenically immunosuppressed patients are slightly younger (median 63). It has been described in association with a variety of immunosuppressive drugs including methotrexate, cyclosporin A, azathioprine, mycophenolate, TNF inhibitors, tacrolimus and topical steroid treatment [46].

#### 2.3.1. Clinical Features

**Figure 3:**
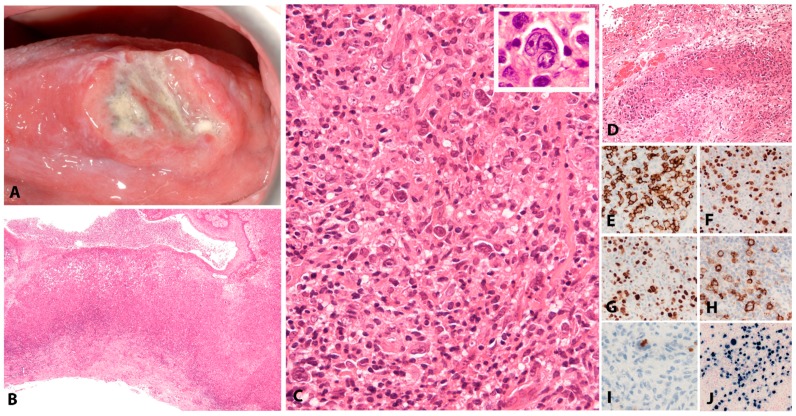
EBV positive mucocutaneous ulcer. (**A**) EBV MCU on the tongue—a shallow ulcer with raised edges and necrotic debris in the centre. The clinical suspicion is often one of squamous cell carcinoma. (**B**) The ulcer is well circumscribed and at the base shows a rim of darker staining small lymphocytes (H&E, 10×). (**C**) On higher magnification, it comprises a polymorphous mixture of lymphoid cells of variable sizes, many with HRS-cell features (H&E, 200×) (inset) in a lymphohistiocytic background (H&E, 600×). (**D**) Angioinvasion is frequently seen. (**E**) The lesional cells are significantly positive for CD20, which highlights variable cell size. (**F**) There is strong expression of PAX5. (**G**) OCT2 is also strongly positive. (**H**) Most cells express CD30. (**I**) There is frequent co-expression of CD15 (immunohistochemistry, D, 100×; E, F, G, H, 200×; I, 400×). (**J**) EBER is abundantly positive, highlighting variability in nuclear sizes of the lesional cells (*in-situ* hybridization, 200×).

EBV+ MCUs are sharply circumscribed, isolated, indurated ulcers in the oropharyngeal mucosa (tonsils, tongue, buccal mucosa, palate), the skin and the gastrointestinal tract (Figure 3A,B). Apart from those related to the ulcer, the patients are otherwise without systemic symptoms, lymphadenopathy, organomegaly or bone marrow involvement. In patients without known immunosuppression, the course is usually waxing and waning. The lesions may be locally troublesome and sometimes locally quite destructive, particularly in instances where iatrogenic immunosuppression is maintained or increased. Without exception, once immunosuppression is withdrawn, full remission is achieved within approximately 8 weeks. Persistent and symptomatic cases in elderly patients who are not additionally immunosuppressed may occasionally require a therapeutic intervention. Rituximab as a single agent achieves excellent results. A very small minority of cases requires further escalation of treatment [46].

#### 2.3.2. Morphology

The infiltrate underlying the ulcer is a polymorphic mixture of plasma cells, lymphocytes, histiocytes, and eosinophils, with scattered large transformed atypical immunoblasts with HRS–like morphology (Figure 3C). Focal necrosis, in addition to surface ulceration, can be present. Angioinvasion is also frequently noted (Figure 3D). Histological distinction from CHL may be difficult. A diagnosis of CHL in the skin or mucosa should be rendered with extreme caution. The lesion originates from the EBV-transformed post-germinal centre B-cells. These large transformed cells are at least partially positive for CD20 but express PAX5 and Oct-2 and are of non-germinal centre phenotype (IRF4/MUM1+, CD10−, BCL6−) (Figure 3E–G). CD30 is positive and CD15 is co-expressed in around half of the cases (Figure 3H,I). LMP1 is mostly co-expressed with EBER in the cells with HRS-like morphology (Figure 3J). The background T-lymphocytes are a mixture of CD4-positive and CD8-positive cells, with a distinctive rim at the base of the lesion [46].

#### 2.3.3. Pathogenesis and Molecular Findings

EBV+ MCU occurs in settings of defective surveillance over EBV due to immunosenescence of advancing age, iatrogenic immunosuppression for autoimmune diseases, solid organ transplantation and HIV infection. The pathogenetic setting, therefore, is similar to EBV+ DLBCL and that seen in PTLD. At least in the elderly, reduced T-cell repertoire and functionality likely play a pathogenetic role. In a recent study of ipilimumab associated colonic ulcers, which were sequentially biopsied, EBV+ MCU developed at the ulcerated areas, which initially did not contain EBV positive lymphoid infiltrate, indicating that these lesions generate a localized environment of “immune sequestration”, leading to locally restricted EBV driven B-cell proliferation [47]. Less than 50% of EBV+ MCUs show clonal IG gene rearrangements. An oligoclonal or restricted TRG pattern is common, reflecting diminished T-cell repertoire [46].

### 2.4. Diffuse Large B-Cell Lymphoma Associated with Chronic Inflammation

DLBCL-CI is a rare EBV associated entity described first in patients with a long history of pyothorax following artificial pneumothorax as treatment for pulmonary tuberculosis [48]. It was included in the 2008 WHO classification as “Pyothorax associated diffuse large B-cell lymphoma” [23]. More recently, lymphomas with the same biological and pathological features have been described in other cavities and confined anatomical spaces in association with chronic inflammation. The same pathogenetic mechanism was postulated and in the 2016 WHO classification, they are now grouped as DLBCL-CI [13]. Most cases published so far are from Japanese patients, but it has occasionally been described in the Western population [49,50,51]. At presentation, the patients are 65–70 years old with a marked male preponderance (12:1). There is usually a very long preceding history of pyothorax (median 37 years) [48,52,53]. DLBCL-CI has also been described in association with chronic osteomyelitis, intrauterine contraceptive device, metallic implants, surgical mesh and chronic venous ulcers, with a preceding history of underlying chronic inflammatory process of more than 10 years [54,55,56].

#### 2.4.1. Clinical Presentation

The pyothorax-associated cases present with chest pain, cough, fever, dyspnoea and swelling. Patients with involvement of other anatomical sites present with local painful swelling. Radiological studies reveal a tumour forming mass with variable invasion of adjacent structures [52,53,57]. This feature distinguishes DLBCL-CI from primary effusion lymphoma (PEL) and FA-DLBCL. DLBCL-CI pursues an aggressive clinical course with a five-year survival of approximately 30%. Interestingly, surgery appears to represent an effective treatment modality for low stage cases. The clinical disease parameters such as poor performance status, advanced stage and high LDH are indicators of poor prognosis [52,53,57].

#### 2.4.2. Morphology

DLBCL-CI displays histological features, which are essentially the same as those of conventional DLBCL. The phenotype is of post-germinal centre B-cell lineage and CD30 is often positive. Plasmablastic differentiation is characterized by CD138 and MUM1 positivity and reduced expression of CD20 and CD79a [51,52]. Aberrant expression of a range of T-cell markers might occur [58]. EBV infection in these cases shows latency III [51,56].

#### 2.4.3. Pathogenesis and Molecular Findings

DLBCL-CI arises as EBV driven lymphoproliferation in a setting of “local immunodeficiency” due to suppuration or inflammation in confined anatomical spaces. This results in a relative segregation of the affected site from the immune system [54,55]. In addition, the lymphoma itself most likely diminishes immunological surveillance by production of IL10 and promotes growth by autocrine IL6-IL6R interaction [59]. The IG genes are clonally rearranged and hypermutated [60]. There is a high degree of genetic complexity [61]. The most common genetic abnormalities include *P53* mutation (70%), MYC amplification and *TNFAIP3* deletion [62,63,64]. The gene expression profiles are distinctively different from those of conventional DLBCL and characterized by signatures related to cellular responses to EBV [65].

### 2.5. Fibrin Associated Diffuse Large B-Cell Lymphoma

FA-DLBCL is a rare EBV+ B-cell lymphoproliferation, which has been included as a provisional entity in the 2016 WHO classification [13]. Some of its features overlap with DLBCL-CI. However, younger patient age, indolent clinical behaviour, excellent survival, as well as histological differences are emerging as distinctive [66]. FA-DLBCL is a proliferation of pleomorphic, large monoclonal B-cells of post germinal centre phenotype in avascular fibrin masses, blood clots, cysts or in vicinity of prosthetic devices. Interestingly, many clinical aspects of FA-DLBCL are similar with those of breast implant-associated anaplastic large cell lymphoma, residing at the interface between reactive lymphoproliferations and neoplasia. Forty-four cases have been published so far, most in the Western population [67,68]. Patients present at a median age of 55 with a male predominance of 3:1 [66].

#### 2.5.1. Clinical Presentation

FA-DLBCL is seen in association with cardiac atrial myxomas (31%), endovascular graft thrombi and chronic haematoma (18%), within a variety of pseudocysts (28%) and adjacent to implanted prosthetic devices (23%) [66,67,68,69,70]. The symptoms are not associated with the lymphoid proliferation but to the underlying condition. Importantly, the lymphoid tissue does not form masses by itself. The patients do not have evidence of lymphadenopathy, organomegaly or other findings to suggest a diagnosis of malignancy. Prognosis, including that of patients treated with surgery only, is excellent. Relapses are seen within the same underlying fibrinous lesion (e.g., within thrombi associated to prosthetic cardiac valves) but prognosis of these patients remains excellent [66].

#### 2.5.2. Morphology

**Figure 4:**
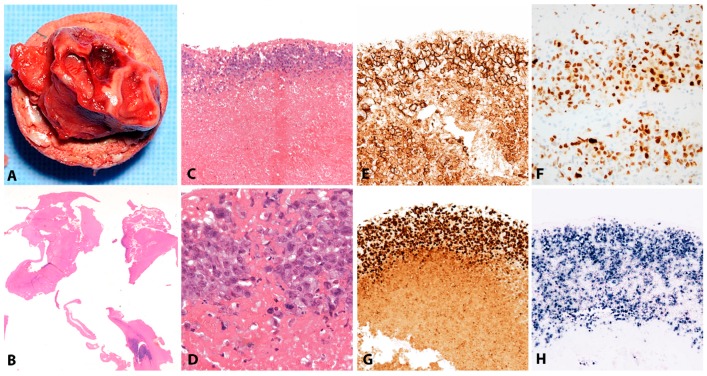
Fibrin associated diffuse large B-cell lymphoma. (**A**) Prosthetic mitral valve with fibrinous vegetation. (**B**) The sections of the fibrin clot show blue staining areas representing cellular lymphoid proliferation (H&E, 5×). (**C**) The lymphoid proliferation forms a band underneath the surface of the clot (H&E, 40×). (**D**) It is composed of large pleomorphic lymphoid cells with focal caryorrhexis (H&E, 400×). (**E**) There is strong expression of CD20 and (**F**) MUM1. (**G**) Ki67 highlights high proliferation (immunohistochemistry, E, F, 100×; G, 40×). (**H**) All cells are EBER positive (*in-situ* hybridization, 40×).

Large, pleomorphic lymphoid cells form a band underlying the surface as well as ribbons and clusters within the avascular substance of the fibrinous thrombi, in atrial myxomas or in the degenerate cellular debris of pseudocysts. Mitotic figures and apoptosis are often seen. The pleomorphic lymphoid cells do not infiltrate any adjacent tissue structures. Unlike DLBCL-CI, a background inflammatory infiltrate is not seen (Figure 4A–D). The immunophenotype is of light chain restricted post germinal centre B-cells (CD45+, CD20+, CD79a+, PAX5+, BCL6+/−, MUM1+) with high expression of CD30. Proliferation fraction on Ki67 immunostaining is more than 90%. BCL2 is positive and there is variable expression of MYC (<50%) and p53 (<30%). All lesional cells are positive for EBER with a latency III phenotype (Figure 4E–H) [66,67,69].

#### 2.5.3. Pathogenesis and Molecular Findings

The universal presence of EBV indicates its important role in pathogenesis. The EBV infected, transformed cells proliferate in the avascular matrix of myxoma, fibrin thrombi or necrotic debris, devoid of inflammatory infiltrate. These features support a pathogenetic hypothesis in which EBV infected B-cells are transformed and proliferate in an “immune privileged” environment due to absence of EBV specific cytotoxic T cells. This is similar to the conditions of cultured B-cells which undergo spontaneous lymphoblastoid transformation by EBV, once T-cells are removed. However, the transformed B-cells, while capable of growth within the immune privileged environment, remain subject to successful immunological surveillance and elimination once they leave the “shielded” space. The tumour cells show monoclonal IGH rearrangements. The small number of cases tested shows a very low level of genetic abnormalities [66].

### 2.6. Lymphomatoid Granulomatosis

LyG is a rare EBV+ B-cell lymphoproliferation described by Liebow et al. in 1972 [71]. He introduced the term “granulomatosis” to distinguish it from Wegener’s granulomatosis of lungs, which overlapped with LyG in its clinical and radiological presentation. The term remained in use even though LyG is not characterized by granulomatous inflammation. Guinee et al. in 1994 demonstrated EBV in the lesional cells and postulated its involvement in pathogenesis [72]. While it still remains a clinicopathological entity in the 2016 WHO classification, there are significant overlapping features with other immunodeficiency related EBV+ B-cell lymphoproliferations [13]. There is some uncertainty amongst the experts as to whether LyG actually represents an entity of its own or is part of the spectrum of EBV positive B-cell LPDs [73]. It is believed to be associated with at least some degree of inherent immunosuppression, and has been described with various types of immunodeficiencies [74,75]. Patients present worldwide in their 30’s–60’s (median 46) with a male preponderance of 2:1 [73].

#### 2.6.1. Clinical Presentation

**Figure 5:**
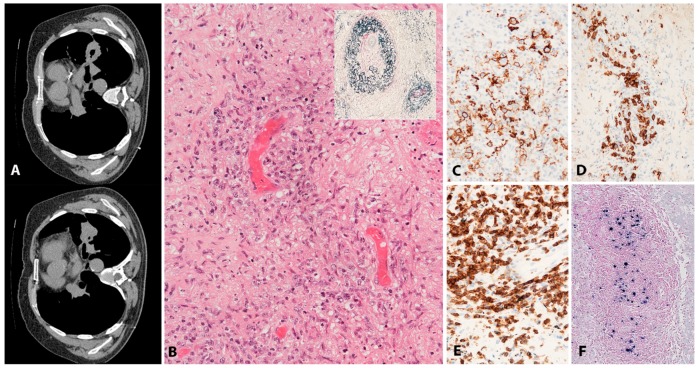
Lymphomatoid granulomatosis. (**A**) Chest CT shows nodular cavitating lesions. (**B**) There is an angiocentric and angioinvasive infiltrate of variably sized lymphoid cells some with Hodgkin-like features (H&E, 100×). The extent of vascular involvement is highlighted by the Elastic-Van Gieson stain (inset) showing the same vessels and consumption of the full thickness of the vascular wall (EVG, 100×). (**C**) CD20 highlights variably sized lesional B-cells including those with Hodgkin-like features (grade II). (**D**) These cells are CD30 positive and follow the contour of the vessel. (**E**) The background lymphoid infiltrate comprises abundant small T-cells highlighted by CD3 (immunohistochemistry, C, 200×; D, E, 100×). (**F**) The lesional B-cells are positive for EBER, which highlights variation in nuclear size. (*in-situ* hybridization, 100×).

The most common and classical clinical presentation is with lung involvement resulting in cough, dyspnoea and pain. Radiologically, there are usually multiple, variably sized bilateral cavitating nodules in mid and lower lung fields (Figure 5A). There may be concomitant involvement of the CNS, skin, liver or kidney. Patients with CNS lesions develop symptoms of confusion, dementia, ataxia, paresis, seizures or cranial nerve signs. Presentation with extrapulmonary lesions only is recognized but is rare and in these circumstances diagnosis should be approached with caution. In particular, cutaneous involvement alone is not seen [73]. Likewise, there is no lymph node and spleen involvement. The disease shows a wide range of clinical behaviour from indolent to aggressive disease [76]. Historical studies show a poor overall survival (40%) of one year [76]. More recent studies show an overall improved progression-free survival (PFS) of 56% in patients with LyG grade 1–2 and PFS of 44% in patients with LyG grade 3 [74,77].

#### 2.6.2. Morphology

Histologically, there is an angioinvasive and angiodestructive lymphoproliferation of variably numerous EBV+ B-cells in a background of small lymphocytic T-cell infiltrate. Grading is based on the numbers of EBV+ B-cells and their morphology. For prognostication and therapeutic decisions, it is important to make a clear distinction between grades 1–2 and grade 3. Grade 1 is characterized by <5, mostly small EBV+ B-cells/HPF, in an abundant T-lymphocytic infiltrate. In grade 2, large but still sparse EBV+ B-cells (5–20/HPF) are easily observed in a background rich in small T cells. The B-cell blasts may form small aggregates (up to 50/HPF) and display HRS-like morphology. In grade 3, the features are of a large B-cell lymphoma, still with rich lymphocytic background. There are numerous EBV+ large B-cells (>50/HPF), many with HRS-like morphology, forming clusters. Due to the striking angioinvasion and arterial obliteration and destruction, LyG is characterized by prominent necrosis. Importantly, granulomas are not observed (except for skin lesions). The tumour cells are CD20+, usually CD30+ but negative for CD15. The character of the background infiltrate is important for differential diagnosis. It is composed of small T-cells (CD3+, CD4+ > CD8+). Unlike CHL, the infiltrate in LyG is much less polymorphous and is devoid of eosinophils and plasma cells. The EBV latency type can be II or III (Figure 5B–F) [13]. Cutaneous lesions of LyG require a special mention as they deviate from the histology seen in the lungs and other sites. They are characterized by subcutaneous panniculitis with non-necrotising granulomas. EBV+ B-cells are much less frequently seen in comparison to lung lesions [73,75]. It has been postulated that cutaneous manifestations may represent an epiphenomenon because of an EBV-induced cytokine effect [73,78].

#### 2.6.3. Pathogenesis and Molecular Findings

Pathogenesis is not well understood. LyG is believed to be associated to at least some degree of inherent immunosuppression and has been described with Wiscott-Aldrich syndrome, X-linked lymphoproliferative syndrome and immunosuppression in transplant patients, HTLV and HIV infection [72,74,75]. Even in patients with no known or clinically evident immunodeficiency, reduced immune functions by laboratory investigations could be seen [74]. EBV latency III indicates an EBV driven B-cell lymphoproliferation arising from ineffective T-cell surveillance. However, some patients show EBV latency II. It has recently been proposed that increased numbers of FOXP3 positive T-cells in grade 3 LyG may play a role in the pathogenesis of disease progression by suppressing tumour specific T-cell immunity. LyG shows variable IGH rearrangements, with a higher rate of monoclonality in grades 2–3. Alterations of oncogenes have not been detected [73].

## 3. Other B-Cell Lymphomas That Can Be EBV-Associated

### 3.1. Plasmablastic Lymphoma

Plasmablastic lymphoma was first described in 1997 in young male patients with HIV infection, involving the jaw and oral cavity [79]. It has subsequently been shown in association with other types of immunodeficiencies including iatrogenic immunosuppression for organ transplantation or autoimmune disorders and in age-related immunosenescence [80,81,82,83]. While the majority of cases are EBV positive, in up to 30% EBV is not detected [82]. PBL is a distinct and aggressive form of NHL defined as a diffuse proliferation of large neoplastic cells mostly resembling immunoblasts, with an immunophenotype of terminally differentiated B-cells characterized by loss of B-cell antigen expression [84,85]. It occurs at all ages with a male preponderance of 4–5:1. Patients with HIV-related disease are younger (median 42) than those with PTLD (median 62) or other HIV negative disease (median 55). PBL is also seen in children, mostly those infected with HIV but also due to other causes of immunosuppression [86,87]. HIV-associated PBL is considered an AIDS-defining illness, and constitutes approximately 3% of HIV-related lymphomas [12,88].

#### 3.1.1. Clinical Features

Patients with PBL usually present with an extranodal mass. HIV-associated PBL has a predilection for the oral cavity (50%). In 45% of the cases there are extra-oral presentations, involving the gastrointestinal tract, sinonasal cavity, skin, soft tissue, lung and bones. In HIV associated cases, lymph node involvement is uncommon, however, in patients with post-transplant PBL lymph nodes are involved in 30% of cases [82,86] Most post-transplant and HIV positive patients (50–75%) present with advanced stage (III/IV). The prognosis is generally poor with a high mortality at a median of 6 to 7 months. Limited stage, outside the HIV context, seems to be associated with much better survival. The EBV positive cases and those expressing CD45 seem to have a better prognosis [81].

#### 3.1.2. Morphology

**Figure 6:**
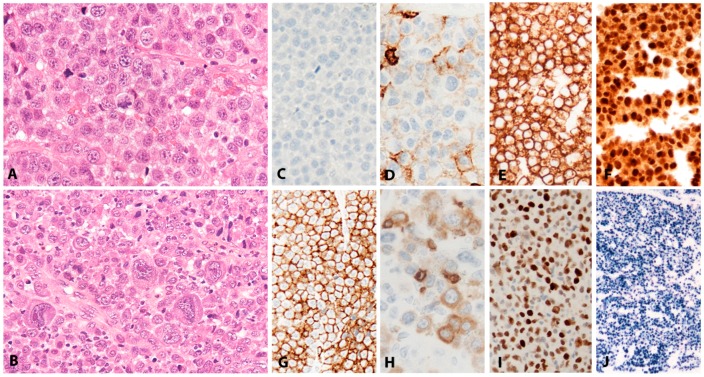
Plasmablastic lymphoma. (**A**) Tumour cells show immunoblastic and some plasmacytic features (H&E, 200×). (**B**) Some tumours show a greater degree of pleomorphism and more marked plasma cell differentiation (H&E, 200×). (**C**) There is lack of expression of CD20 and (**D**) CD45. (**E**) The tumour cells are positive for CD138 and (**F**) MUM1. (**G**) There is strong expression of CD56 in some tumours. (**H**) Aberrant expression of CD3 may be observed. (**I**) There is significant positivity for MYC (immunohistochemistry, C–G, I 200×, H, 400×). (**J**) All tumour cells are positive for EBER (*in-situ* hybridization, 100×).

PBL shows a range of morphological features ranging from diffuse proliferation of monomorphic immunoblast-like cells to cells with prominent plasmacytic differentiation. A “starry sky” pattern due to high mitotic activity, abundant apoptotic bodies, and tingle body macrophages is common in the monomorphic cases (Figure 6A,B). Geographic necrosis may also be seen. The phenotype is that of terminally differentiated B lymphocytes. The neoplastic cells lack expression of B-cell specific markers (CD19, CD20, PAX5) and show expression of plasma cell markers (CD79a, IRF4/MUM1, BLIMP1, CD38 and CD138) as well as cytoplasmic immunoglobulin. BCL2 and BCL6 are usually not expressed, whereas CD10 is expressed in up to 32% of cases and seems to correlate with *MYC* translocation [82,85]. Epithelial membrane antigen and CD56 are detected in up to 27% of the cases. Ki-67 staining reveals a high proliferation rate (>90%). MYC protein seems to be overexpressed in all cases [85], however, 69% of the cases carry *MYC* translocations or amplification (Figure 6C–I) [89]. The co-expression of MYC and BLIMP1 was recently reported in 80% of PBLs [85]. PBL shows a latency I or II pattern of expression of EBV associated markers. EBER is positive in 70–80% of the cases and LMP1 might be positive in few cells (Figure 6J).

#### 3.1.3. Pathogenesis and Molecular Findings

The pathogenesis of PBL involves the HIV related factors together with EBV infection. However, EBV is negative in 30% of cases and alternative pathogenetic mechanisms may be involved. PBL has clonally rearranged immunoglobulin genes, which vary in their hypermutation status. Genetic studies have identified complex karyotypes with frequent *MYC* translocations (50%) [89]. The EBV positive cases are more frequently associated with *MYC* alterations when compared to the EBV negative ones (74% vs. 43%). It is believed that the activation of *MYC* may be important for the pathogenesis of the disease to overcome the repressor effects of BLIMP1, and provide the tumour cells with a proliferative and survival advantage. Interestingly, MYC overexpression is not restricted to cases with genetic alterations of *MYC* (translocation and amplification), indicating that there are other epigenetic mechanisms that deregulate MYC expression [85]. *PRDM1* somatic mutations targeting critical functional domains involved in the regulation of *MYC* were recently identified in 49% of PBLs [85]. The authors suggested that the aberrant co-expression of MYC and PRDM1/Blimp1 is responsible for the phenotype of PBL. Of note, recent gene expression studies have revealed that neither the presence of EBV nor HIV infection affect the transcriptome profile of PBL, indicating that both HIV-associated and age-related PBLs are defined by the same phenotype [90].

### 3.2. Burkitt Lymphoma

Burkitt lymphoma represents a historical paradigm in terms of characterization of a neoplasm from clinical and histological observations through to understanding of viral and genetic basis of oncogenesis. In 1958 Dennis Burkitt described the clinical features of a cancer in Equatorial African children and Dennis Wright provided the histological characterization of the lymphoma credited with Burkitt’s name [91,92]. BL is a highly aggressive B-cell neoplasm characterised by *MYC* rearrangement and variable EBV involvement. There are three epidemiological types of BL. This diverse landscape implicates potentially different pathogenetic mechanisms and variable involvement of EBV. “Endemic” BL (eBL) is seen in parts of the world with high prevalence of early EBV infection and endemic P. falciparum malaria (Equatorial Africa and Papua New Guinea), where it represents the most common malignancy in children. “Sporadic” BL (sBL) occurs throughout the world, representing up to 2% of all lymphomas in the Western population. It is seen at all ages (median 30 years), and is particularly common in children (up to 50% of childhood lymphomas) but also affects elderly patients. Association with EBV is variable, being much higher in South Africa and South America (80%) but low in Europe and the USA [93,94,95]. The “Immunodeficiency associated” BL (iBL) is particularly associated with HIV infection, but can be seen in other immune deficient states. Approximately 30–40% are EBV positive. In comparison with the healthy population, there is a 100-fold increased risk of developing iBL in HIV patients early in the course of the infection. This has not changed with the introduction of HAART [96].

#### 3.2.1. Clinical Features

Clinical presentation of BL differs between the three epidemiological types. sBL presents with extranodal disease as intra-abdominal masses, involving ileo-caecum, omentum, ovaries, kidneys or breasts (often bilaterally). Jaw and facial bone involvement seen as the most common presentation in eBL is not a feature of sBL. Bone marrow is variably involved. In contrast, iBL presents with nodal disease, common marrow and frequent CNS involvement. Leukemic phase may be part of the clinical presentation. A purely leukemic variant exists but is rare [13]. BL is characterised by an exceptionally fast growth, hence, duration of symptoms until patients present with bulky disease is short and in the range of several weeks, at most. With chemotherapy, current survival of BL reaches 70–90%. Survival is significantly better in children. High disease burden at presentation including tumour masses greater than 10 cm and high LDH, together with CNS involvement, represent adverse prognostic parameters [13].

#### 3.2.2. Morphology

**Figure 7:**
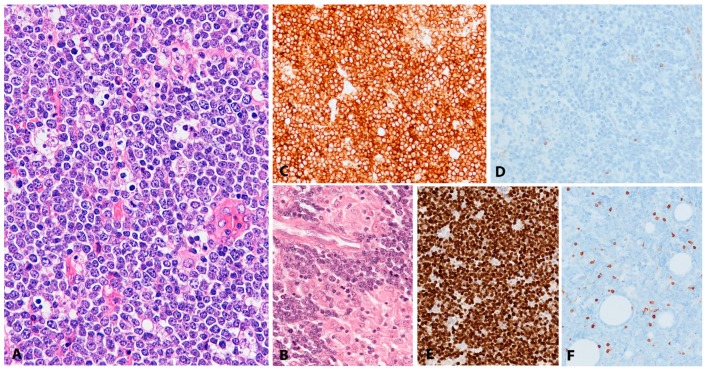
Burkitt lymphoma. (**A**) The tumour is composed of regular, medium size cells with basophilic cytoplasm, coarse nuclear chromatin and small peripheral nucleoli; tangible body macrophages are scattered through the tumour generating a starry sky appearance (H&E, 100×). (**B**) Some tumours show copious granulomatous reaction, which may obscure the lymphoma. (**C**) There is strong, uniform expression of CD10. (**D**) No expression of BCL2 is seen. (**E**) Ki67 shows 100% proliferation fraction. (**F**) CD3 shows very occasional reactive small T cells (immunohistochemistry, B–F, 100×).

Histologically, BL is composed of a diffuse growth of uniform medium-sized lymphoid cells with basophilic cytoplasm and round regular nuclei with coarse chromatin and peripherally placed small nucleoli. In cytological preparations, cytoplasmic vacuoles are observed. There is high mitotic activity and marked apoptosis with tingible body macrophages, resulting in a “starry sky” appearance (Figure 7A). BL is devoid of background reactive infiltrate; however, granulomatous reaction is occasionally seen to the extent that it may obscure the lymphoma (Figure 7B,F). BL with granulomatous reaction is EBV-associated and these cases tend to undergo spontaneous remission [97]. Greater nuclear irregularity and plasmacytoid features could be seen in iBL, which has in the past presented diagnostic and classification problems. Gene expression profiling clusters these cases with the rest of the BLs [98]. BL expresses strongly surface IgM, the full spectrum of B-cell lineage markers (CD19, 20, CD22, CD79a and PAX5) and a germinal centre phenotype (CD10, BCL6), importantly, lacking expression of BCL2 (Figure 7C,D). There is strong, uniform nuclear expression of MYC in all tumour cells and 100% proliferation by Ki67 immunostaining (Figure 7E) [97].

#### 3.2.3. Pathogenesis and Molecular Findings

The pathogenetic role of the *MYC* gene and EBV infection in BL has been extensively studied over the past 5 decades [3,99]. However, despite wealth of acquired information, the pathogenetic mechanisms are not yet completely understood. The *MYC* gene encodes a transcription factor, which controls transcription of 10–15% of human genes, stimulating cell proliferation and growth [100]. In BL *MYC* is activated through a translocation to one of the immunoglobulin gene promoters. However, this alone is insufficient for independent tumour growth, as *MYC* induced *P53* activation results in apoptosis, counterbalancing the *MYC* proliferative effect. Concomitant *P53* mutations seen in 30% of BLs are believed to be responsible for suppression of apoptosis and it is suggested that epigenetic control of *P53* through *P19/ARF* and *MDM2* plays a significant role facilitating *MYC* driven proliferation [101]. In addition, *MYC* itself is mutated in BL making it less potent in activating *P53* mediated apoptosis.

Existence of different epidemiological types variably associated with EBV suggests its non-essential pathogenetic role, and potential alternative pathogenetic pathways. Within the tumour cells the EBV itself is clonal, indicating infection prior to the establishment of the neoplastic clone. However, it is uncertain whether EBV infection precedes or follows *MYC* translocation. In this respect, there are two possible scenarios of pathogenetic EBV involvement. In the first, the viral genes facilitate cell survival after *MYC* rearrangement has taken place by abrogating apoptosis through the activity of oncogenic EBERs, EBNA1 or BART-mRNAs and their interactions with anti-apoptotic proteins such as survivin or *P53* regulator *USP7* [102,103,104,105]. In addition, LMP2 has been shown to activate the PI3K pathway contributing to persistent B-cell receptor (BCR) signalling and thus aiding *MYC* driven lymphomagenesis [106,107,108]. In the second scenario, EBV promotes *MYC* rearrangement through EBNA3C mediated induction of activation-induced cytidine deaminase (AID), and/or by EBNA2 facilitated increased susceptibility of the *MYC* enhancer region to AID induced breaks [109,110]. Plasmodium falciparum has also been found to induce AID and may play a synergistic role in pathogenesis of eBL [111]. Relevant for either of the scenarios is that LMP2A, EBNA2 and EBNA3C are transcribed in latency III, while BL has historically been considered the prototype of EBV latency I neoplasm. However, recent evidence indicates significant latency fluidity in BL making the above pathogenetic events entirely plausible [112,113].

BL originates from germinal centre B-cells and has clonally rearranged IG genes which are hypermutated. At the molecular genetic level, the IGH*/MYC* breakpoints differ significantly between eBL and sBL, suggesting that they arise at different stages of cell differentiation and maturation, respectively [114,115]. The NGS studies have identified a spectrum of mutated genes in 5–40% of BL including those controlling cell cycle, apoptosis and chromatin remodelling [112,116,117,118,119]. Importantly, there are significant differences between eBL and sBL in their mutational landscape. In sBL, 70% are affected by mutations in *TCF3* or its negative regulator *ID3*. *TCF3* is a master regulator of genes in proliferating centroblasts. Its activation results in BCR independent signalling activating the PI3K/ATK pathway, which acts synergistically with *MYC* and promotes cell survival. Only 30% of eBLs have the same mutations [107,112,115]. In addition, there is an inverse correlation between the mutational rates and EBV positivity between eBL and sBL. This overall suggests that there are indeed different pathogenetic mechanisms in the development of the epidemiological subtypes of BL. Thus, in sBL increased mutational frequency may supplement the pathogenetic effect of the virus in eBL in facilitating persistent BCR signalling to promote cell survival [112,119]. Approximately 10% of BLs with typical histology and immunophenotype show no evidence of *MYC* gene rearrangement. In some of these cases this may be a result of technical inability of the currently available FISH probes to hybridize to rearrangements outside the major breakpoint cluster. In others, *MYC* may be upregulated by DNA hypermethylation mediated epigenetic mechanisms or EBV-encoded microRNA’s [120,121]. Some of these *MYC* rearrangement negative lymphomas may represent the recently described Burkitt-like lymphoma with 11q alteration [122].

### 3.3. Classic Hodgkin Lymphoma

In addition to the entities described above, EBV is associated only with a subgroup of CHL cases, indicating that the virus is a non-obligatory oncogenic factor for this disorder. In terms of absolute numbers, EBV+ CHL is probably the most common EBV-related lymphoma worldwide, and was one of the first lymphomas for which EBV-association was suspected and subsequently proven [123]. The frequency of EBV in CHL ranges from less than 20% in nodular sclerosis subtype in Western countries to virtually 100% in childhood cases from developing countries and immunosuppressed hosts [124]. These differences indicate a significant influence of ethnicity, age, immune and socio-economic status. CHL, which accounts for 15–25% of all lymphomas, is unique in several aspects, and therefore, is separated from the NHL in the WHO classification.

#### 3.3.1. Clinical Features

Patients with CHL usually present with lymphadenopathy, most commonly involving only one or two areas in the cervical region. Up to 40% of the patients have B symptoms characterized by night sweats, fever and significant weight loss. Mediastinal involvement is most frequently seen with nodular sclerosis subtype whereas liver and spleen involvement are more common with mixed cellularity subtype. The prognosis has improved with modern multimodal therapy with cure rates of 80–90%.

#### 3.3.2. Morphology

**Figure 8:**
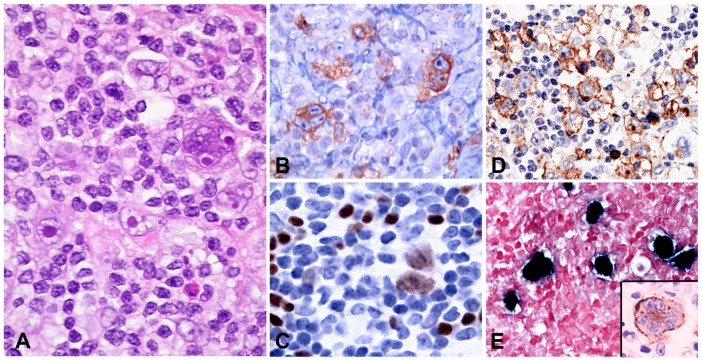
Classic Hodgkin lymphoma (CHL). (**A**) Lymph node infiltrated by CHL with Hodgkin and Reed-Sternberg cells (HRS cells) in a mixed reactive background composed of eosinophils, small lymphocytes, histiocytes and plasma cells (H&E, 400×). (**B**) CD30 highlights the HRS cells. (**C**) The HRS cells are weak PAX5 positive, in contrast to the small reactive B cells, which show strong PAX5 nuclear staining. (**D**) CD15 is positive in the HRS cells (immunohistochemistry, B–D, 400×). (**E**) EBERs *in-situ* hybridization is only positive in HRS cells (400×). Inset: EBV infected cell expresses the EBV-encoded latent membrane protein 1 (LMP1) (immunohistochemistry, 400×).

CHL is characterized by a minority of large atypical, neoplastic cells called HRS cells in a strong inflammatory background (Figure 8A). The tumour cells are clonal germinal centre B cells, which have down regulated the B-cell expression program [125,126,127]. Therefore, the tumour cells lack expression of most B-cell markers and transcription factors OCT2 and BOB1 [128]; however, the weak expression of PAX5 confirms the B cell origin of the tumour cells (Figure 8C) [129]. The HRS cells are characterized by strong and homogeneous positivity for the CD30 antigen, MUM1 and frequently CD15 (Figure 8B–D) [13]. CHL EBV-associated shows constant expression of LMP1 protein [130,131], and therefore is the prototypic EBV-associated disorder with latency II (Figure 8E). In contrast to other LPDs, LMP1 immunostaining can be used as a reliable marker for EBV-association. A characteristic feature, useful for diagnosis, is that in CHL only the HRS cells are EBV infected without small reactive lymphocytes.

#### 3.3.3. Pathogenesis and Molecular Findings

EBV has been postulated to play a role in the pathogenesis of CHL. In CHL associated with EBV, the virus is present in clonal episomal form, indicating its presence from the start of neoplastic transformation [132]. Oncogenic LMP1 signalling seems to be an important factor in the survival of the neoplastic cells, which otherwise would undergo apoptosis in the germinal centre due to their lack of a functioning B-cell receptor [133]. LMP1 mimics activated CD40, a member of the tumour necrosis receptor superfamily, and leads to constitutive activation of NF-kappaB and JAK/STAT signalling pathways in HRS cells, inducing proliferation and survival [134]. LMP2A may replace B-cell receptor signalling in CHL, essential for the survival of growth-transformed germinal centre B cells [135,136]. The fact that cells expressing the strongly immunogenic LMP1 protein can survive in an immunocompetent host points to a profound deregulation of the local immune response [137,138]. The neoplastic cells secrete a variety of cytokines and chemokines, resulting in an immunosuppressive microenvironment dominated by T-helper cells type 2 (TH2), and exhibit a variety of immune evasion mechanisms, such as expression of PD-1 and PD-1 ligands and Galectin-1, the former representing a novel target for therapy [139,140,141]. Although clinically and pathologically EBV+ and EBV-CHL are very similar, indicating that the main impact of EBV infection is in the early stages of disease development, some important differences have been observed. The so-called “crippling” mutations of the rearranged IGH genes preventing any immunoglobulin transcription are virtually exclusive to EBV+ cases, whereas both aberrant expression of multiple receptor tyrosine kinases and mutations in the NF-kappaB inhibitor *TNFAIP3* are predominantly found in EBV-cases, indicating distinct pathways to survival and NF-kappaB activation depending on EBV status [125,133,142,143].

## 4. Other B-Cell Lymphomas Rarely EBV-Associated

### 4.1. Chronic Lymphocytic Leukaemia

Infection with EBV is a rare secondary phenomenon in low-grade B-cell lymphomas, and most commonly occurs in chronic lymphocytic leukaemia (CLL). In lymph nodes involved by CLL, EBER and LMP1 positive large cells frequently resembling HRS cells can occasionally be observed. They are set in the background of conventional CLL and do not show prognostic relevance. In rare cases of CLL, true CHL—both clonally related, as well as unrelated—can develop, characterized by the typical T-cell dominated background; these cases are also frequently EBV+, with latency type II [144].

### 4.2. Plasma Cell Myeloma

Plasma cell myeloma, a common neoplasm of terminally differentiated B cells usually presenting with bone marrow infiltration, multiple osteolytic lesions and monoclonal immunoglobulin in serum and/or urine, is usually EBV negative, but recently exceptional cases have been found to show evidence of EBV infection in all neoplastic cells [145]. The clinical relevance of this finding is unknown. Of interest, solitary plasmacytomas of the head and neck region, indolent plasma cell neoplasms with low risk to develop into systemic multiple myeloma have been found to contain EBV in up to 17% of cases, possibly due to their location in the site of EBV primary infection [146,147,148].

The differential diagnosis between EBV+ plasma cell neoplasms and PBL relies on a combination of clinical, morphological and phenotypic characteristics since not a single feature is diagnostic for either entity. PBL occurs predominantly in immunosuppressed hosts, most commonly in the setting of HIV infection and frequently arises in extranodal locations including the oral cavity, whereas bone marrow involvement, M-protein and osteolytic lesions are infrequent, in contrast to EBV+ plasma cell myeloma. PBL exhibits predominantly immunoblastic or plasmablastic cytomorphology. Mature plasma cell morphology is a strong indicator of a plasma cell neoplasm and should discourage a diagnosis of PBL. Both EBV+ plasma cell neoplasms and PBL show significant phenotypic overlap with expression of plasma cell markers and lack of B-cell antigens, but PBL is less common CD56+ and more frequently aberrantly expresses T-cell markers. Nevertheless, rare cases of EBV+ neoplasms in immunosuppressed hosts with clinical features of myeloma exist and may represent a true grey zone between EBV+ plasma cell myeloma and PBL.

## 5. EBV-Associated T and NK-Cell Lymphoproliferative Diseases

EBV associated T- and NK-cell LPD are prevalent in Asia and Native American populations of Mexico, Guatemala, Peru and Bolivia [149,150,151,152,153]. These disorders affect the paediatric population, young adults and elderly patients. The revised 2016 WHO lymphoma classification recognizes the EBV-positive T and NK-cell disorders listed in Table 2. Chronic active EBV (CAEBV) infection is not considered a malignancy but because it might be misdiagnosed as lymphoma has been introduced in the WHO classification to make clinicians and pathologists aware of this disorder. Three disorders are recognized representing the spectrum of CAEBV associated T and NK-cell lymphoproliferative disorder (LPD) with different clinical presentations; one systemic, and two cutaneous disorders (hydroa-vacciniforme-like LPD and severe mosquito bite allergy) [149]. The malignant disorders include systemic EBV-positive T-cell lymphoma of childhood, aggressive NK/T-cell leukaemia, extranodal NK/T-cell lymphoma, nasal type, and the new provisional subgroup within the PTCL, not otherwise specified (NOS) designated primary EBV-positive nodal, T- or NK-cell lymphoma. The main characteristics of these LPDs are summarized in Table 4.

### 5.1. Chronic Active EBV Infection of T and NK Cell Type, Systemic Form

Although most symptoms associated with IM resolve within weeks to months, there can be persistent symptoms and lasting disease that develops following primary EBV infection. This complication is known as chronic active EBV infection (CAEBV). CAEBV was originally described by Straus et al. [154,155], as a disease related to persistent EBV infection lasting longer than 6 months after the acute EBV infection with IM-like symptoms, elevated titres against EBV, and evidence of organ damage without evidence of an underlying immunodeficiency. Although originally considered as an infection targeting B cells, subsequent studies demonstrated that this disorder is more often associated with infection of T cells and less often of NK cells [156]. The revised diagnostic criteria of CAEBV include IM-like symptoms persisting more than 3 months, increased EBV DNA (>10^2.5^ copies/mg EBV DNA) in peripheral blood, histological evidence of organ disease, and demonstration of EBV RNA or viral protein in affected tissues in patients without known immunodeficiency, malignancy or autoimmune disorders [8,157].

CAEBV, systemic form is a polyclonal, oligoclonal or often monoclonal T- or NK-cell lymphoproliferation that exhibits varying degrees of clinical severity depending on the host immunity and EBV viral load. Age at onset (>8 years) and liver dysfunction are risk factors for mortality. It has a strong racial predisposition with most cases reported in Japan [152,156,157], Korea [151] and Taiwan [153]. It has been reported in Latin America and rarely in Western [158] and African populations [159]. It occurs most often in children and adolescents. Adult onset is rare but appears rapidly progressive and more aggressive [160,161]. There is no sex predilection.

#### 5.1.1. Clinical Features

The characteristic IM-like features with prolonged or intermittent fever, hepatosplenomegaly and lymphadenopathy occur only in approximately 50% of the patients. Because the clinical presentation is so variable, the diagnosis is difficult and often delayed. Cutaneous manifestations are common an include hypersensitivity to mosquito bites (33%), rash (25%), and HV-like eruptions (10%). Other accompanying symptoms are diarrhoea (6%) and uveitis (5%) [162]. The clinical course is protracted and many patients survive for many years without disease progression, whereas other patients develop life-threatening complications like hemophagocytic syndrome (24%), coronary artery aneurysm (9%), hepatic failure (15%), interstitial pneumonia (9%), CNS involvement (7%), gastrointestinal perforation (11%) and myocarditis (4%). The clinical course also varies depending on the predominantly infected cell type in the peripheral blood [150,156,163]. Patients with T-cell CAEBV have a shorter survival time than those with NK-cell type disease. The 5-year survival rate for patients with T-cell CAEBV is 59% whereas patients with NK-cell CAEBV have a survival rate of 87%. Patients with T-cell CAEBV often present with prominent systemic symptoms, high titres of EBV specific antibodies and have rapid progression of their disease. In contrast, patients with NK cell disease, in addition to mild systemic symptoms often have hypersensitivity to mosquito bites, rash, high levels of IgE, and do not always have elevated EBV-specific antibody titres. In around 16% of the patients, progression to NK/T-cell lymphoma or aggressive NK-cell leukaemia has been reported [150,151,164]. Laboratory tests reveal pancytopenia and abnormal liver functions. Most patients have high antibody titres of EBV VCA IgG and early antigen IgG, and often IgA antibodies against VCA and early antigen are detected [165]. By definition, all patients have elevated levels of EBV DNA in the peripheral blood.

#### 5.1.2. Morphology

**Figure 9:**
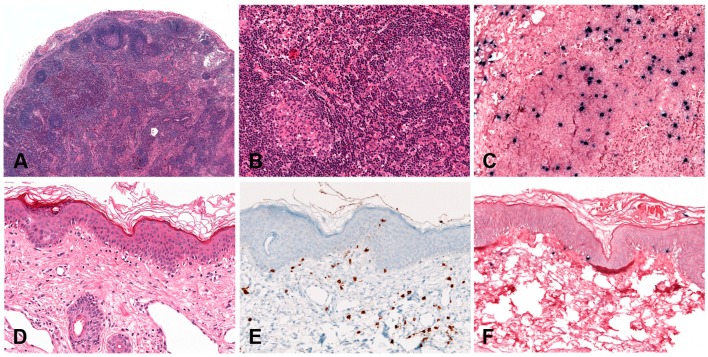
Chronic active EBV infection, systemic form. (**A**) Lymph node with preserved architecture (H&E, 25×). (**B**) Higher magnification demonstrates normal reactive germinal centres (H&E, 200×). (**C**) EBER *in-situ* hybridization shows positive cells both in the follicles and in the interfollicular areas (magnification, 200×). (**D**) Skin biopsy shows a discrete lymphoid infiltrate in the upper dermis and in the sub-epidermis (H&E, 200×). (**E**) CD3 is positive in the lymphocytes (magnification 200×). (**F**) Scattered T cell lymphocytes are EBER positive (*in-situ* hybridization, 200×).

In general, patients with CAEBV do not show changes suggestive of a malignant disorder in the affected tissues (Figure 9A–F). The lymph nodes show variable histological features with paracortical hyperplasia, follicular hyperplasia, focal necrosis and small epithelioid granulomas (Figure 9A–C). A polymorphic infiltrate might be observed. The spleen shows atrophy of the white pulp with congestion of the red pulp. The liver shows portal or sinusoidal infiltration of small lymphocytes without atypia suggestive of viral hepatitis. CAEBV mimics interstitial pneumonitis in the lung and viral myocarditis in the heart [159], and might present with skin rash (Figure 9D,E). The bone marrow usually looks normal. In cases complicated by hemophagocytic syndrome, histiocytic hyperplasia with erythrophagocytosis might be seen in bone marrow, liver and lymph nodes [150]. The immunophenotype of the EBV-infected cells is in the majority of cases of T-cell phenotype (59%) predominantly CD4+; however, CD8+, T-cell receptor genes (TCR)αβ and TCRγδ+ cells have been documented. CAEBV of NK cell type represents around 41% of the cases, and both T and NK cells 4% of the cases [150]. CAEBV of B cell type is a rather rare disease (2%) affecting mainly Caucasian population [166].

#### 5.1.3. Pathogenesis and Molecular Findings

The pathogenesis of this disorder is unknown; however, the strong racial predisposition in immunocompetent individuals suggests an abnormal immune response to EBV most probably due to genetic polymorphisms in genes related to the EBV immune response [8,157]. EBV-specific cytotoxic T-lymphocyte activity is impaired in patients with CAEBV [167,168]. The expression of EBV-related antigens is limited to EBNA1, LMP1 and LMP2. Other EBV-related antigens like EBNA2, 3A or 3B are not expressed [163,169]. Although CAEBV is not considered a neoplastic disorder, monoclonal rearranged TCR are demonstrated in 84% of the cases, oligoclonal in 11% and polyclonal in only 5% [150]. Chromosomal aberrations have been reported only in a minority of the cases [150]. A prognostic classification for CAEBV based on cytology and clonality of the proliferating cells has been proposed. [164] Category A1 is a polymorphic and polyclonal proliferation of T or NK cells, Category A2 is polymorphic and monoclonal, and Category A3 is monomorphic and monoclonal. This classification reflects the continuous spectrum of CAEBV to overt lymphoma. According to the WHO classification, category A3 should be classified depending on the infected cell as EBV+ T- or NK-cell lymphoma.

### 5.2. Hydroa Vacciniforme-Like Lymphoproliferative Disease

Hydroa vacciniforme-like lymphoproliferative disorder (HV-like LPD) is a chronic EBV+ LPD of childhood with risk to develop a systemic lymphoma [170]. It is one of the cutaneous forms of CAEBV. HV-like LPD has been described mainly in children from Mexico [171], Peru [172] and Asia [173,174]. Because earlier studies demonstrated that these lesions were associated to EBV infection [173,175] and often showed monoclonal rearrangement of the TCR genes [172], the term HV-like lymphoma was suggested and incorporated in the WHO 2008 classification [176]. However, due to the broad clinical spectrum of the disease, and the lack of reliable morphological and molecular criteria to predict clinical behaviour—classic HV vs. HV-like lymphoma—the term HV-like LPD has been proposed and incorporated in the 2016 revised WHO classification [149].

#### 5.2.1. Clinical Features

**Figure 10:**
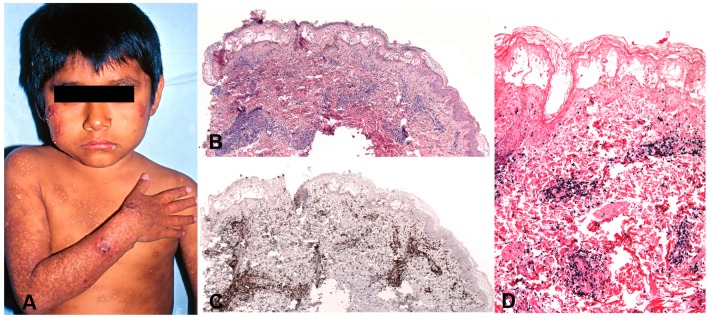
Hydroa vacciniforme-like lymphoproliferative disorder. (**A**) Sun expose areas of face and arms show papulovesicular eruptions with crusts alternating with varicelliform scars after healing. (**B**) Skin biopsy with intraepidermal bullae and a dense infiltrate in the dermis surrounding adnexae and blood vessels (H&E, 50×). (**C**) The lymphoid infiltrate is CD8 positive (magnification 50×). (**D**) The lymphoid infiltrate is positive for EBV, as demonstrated by *in-situ* hybridization for EBV-encoded small RNA (EBER) (100×).

This disorder is seen mainly in children with a median age at diagnosis of 8 years (range: 1–15 years) [170]. It is rare in adults. The severity of the skin lesions and the clinical presentation varies among the patients and shows a broad spectrum [150,177,178]. There is seasonal variation, with increased recurrences in spring and summer. Patients usually present with a dermatosis characterized by vesicles, blisters, erythema, ulcerations, crusts and vacciniform scars involving at the beginning face, ear lobes and hands (sun-exposed areas) but with time the lesions become generalized in both covered and sun-exposed areas (Figure 10A). Light avoidance does not prevent the development of skin lesions. Some cases present with a very indolent course characterized by remissions and recurrences that may finally progress to a more severe disease. In severe cases, the skin lesions are larger and deeper producing extensive tissue loss and disfigurement. Systemic symptoms such as fever, lymphadenopathy and/or hepatosplenomegaly are common in the severe cases. A rare clinical presentation with periorbital swelling has been reported in children from Bolivia [179].

#### 5.2.2. Morphology

The skin biopsies showed similar histological findings, characterized by a lymphoid infiltrate predominantly in the dermis that sometimes extends deep into the subcutaneous tissue [170]. The infiltrate is located around adnexae and blood vessels, often with angiodestructive features. The intensity of the infiltrate and the atypia of the lymphocytes varied from case to case. In many cases an intraepidermal spongiotic vesicle without epitheliotropism is observed (Figure 10B). In more severe cases the overlying epidermis is ulcerated. The infiltrating lymphocytes have mostly a CD8+ T-cell phenotype with few cases being CD4+ (Figure 10C). A third of the cases showed an NK-cell phenotype with expression of CD56 [150,170,180]. In rare cases infection of both T and NK cells has been documented [170]. Interestingly, in most cases a clonal expansion of gamma delta T-cells has been documented in the peripheral blood [181,182] but only in rare cases in the infiltrating lymphocytes in the skin [170,183]. CD30 is often expressed in the infiltrating EBV+T-cells but LMP1 is rarely positive [170].

#### 5.2.3. Pathogenesis and Molecular Findings

The aetiology is unknown but like in other EBV associated LPDs, predisposition for this condition may be related to a defective cytotoxic immune response to EBV. Most cases have clonal rearrangement of the TCR genes [150,170]. T-cell monoclonality does not predict the clinical behaviour of the disease. EBER in-situ hybridization (ISH) is positive but the number of positive cells varies from case to case and sometimes only a small percentage of the infiltrating lymphocytes are EBV positive (Figure 10D). LMP1 is usually positive by PCR in peripheral blood indicating an EBV latency type II [184].

### 5.3. Severe Mosquito Bite Allergy

#### 5.3.1. Clinical Features

Severe mosquito bite allergy is a rather uncommon cutaneous manifestation of CAEBV of NK-cell type, mainly reported in Japan [150,185,186,187]. It is characterized by hypersensitivity to mosquito bites with acute, sometimes severe skin lesions including erythema, bullae, ulcers and necrosis with scarring. During the acute episode, it is frequently accompanied by high fever and general malaise. Patients show high level of serum IgE, high EBV DNA load in peripheral blood and NK-cell lymphocytosis. Occasionally, lymphadenopathy and/or hepatosplenomegaly can occur. After recovering, patients are asymptomatic until the next mosquito bites. This disorder might progress to systemic CAEBV and eventually to aggressive NK cell leukaemia [150,156,188].

#### 5.3.2. Morphology

The morphology is similar to HV-like LPD but the infiltrating lymphocytes show an NK-cell phenotype with expression of CD56, and cytototoxic granules TIA-1 and granzyme B. The infiltrate tends to extend into the subcutaneous tissue. Reactive T-cells of both CD4 and CD8 phenotype are found with different intensities. As in HV-like LPD, CD30 is often positive in the EBV infected cells but LMP1 is rarely positive.

#### 5.3.3. Pathogenesis and Molecular Findings

The aetiology is unknown. The hypersensitivity to mosquito bite is due to CD4+ T cell proliferation in response to mosquito salivary gland secretions that seems to play a key role in the reactivation of EBV in NK cells inducing the expression of LMP1 [189,190,191]. The NK cells are infected with monoclonal EBV demonstrated by terminal repeat analysis [156]. As in HV-like LPD, only a fraction of the NK cells are positive for EBER *ISH*. It is also considered an EBV latency type II disease because LMP1 is detected in peripheral blood by PCR.

### 5.4. Systemic EBV-Positive T-Cell Lymphoma of Childhood

Systemic EBV-positive T-cell lymphoma of childhood and young adults is a fulminant illness characterized by a clonal proliferation of EBV-infected T cells with a CD8+ cytotoxic phenotypem [192]. It occurs shortly after primary acute EBV infection or rarely develops in the clinical setting of CAEBV infection. The disease is prevalent in Asia and Latin America and is nearly always accompanied by hemophagocytic syndrome. In recognition of the aggressive clinical behaviour the term “lymphoproliferative disorder,” introduced in 2008 to the WHO classification [176], was changed to “systemic EBV-positive T-cell lymphoma” in the revised 2016 WHO classification [149].

#### 5.4.1. Clinical Features

Previously healthy Individuals develop characteristic symptoms of infectious mononucleosis with fever, general malaise and upper respiratory illness after primary EBV infection. Within a period of 1 to 3 weeks, patients develop hepatosplenomegaly, liver dysfunction, pancytopenia, rash and central nervous system symptoms. Lymphadenopathy is not common. The disease is usually complicated by hemophagocytic syndrome, coagulopathy, sepsis and multiorgan failure [192]. Despite the acute EBV infection, serology shows low or absent anti-VCA IgM antibodies with positive anti-VCA IgG titres. The abnormal serology in these cases is misleading, which usually delays the diagnosis. Most patients die within days to weeks of diagnosis. Few cases have been reported to respond to an etoposide- and dexametasone based regimen (HLH-2004 protocol) [193,194,195] followed or not by allogeneic haematopoietic stem cell transplantation.

#### 5.4.2. Morphology

Hyperplasia of histiocytes and marked hemophagocytosis with increased numbers of T lymphocytes are the most striking histologic changes in the bone marrow, spleen and liver [192]. The infiltrating T cells are usually small and lack cytological atypia; however, some cases showed pleomorphic medium-sized to large lymphoid cells, irregular nuclei and frequent mitosis [152]. The liver shows mild to prominent portal and/or sinusoidal infiltrates with cholestasis, steatosis and focal necrosis. The spleen shows depleted white pulp with prominent red pulp lymphoid infiltration and striking hemophagocytosis. The lymph nodes usually show preserved architecture with open sinuses. Early in the disease there is expansion of the interfollicular areas with T cells with a broad cytological spectrum ranging from small-sized cells to large atypical lymphocytes with irregular nuclei and abundant cytoplasm. The more advanced the disease the more depleted the lymph nodes look. Bone marrow biopsies show histiocytic hyperplasia with prominent erythrophagocytosis. The infiltrating cells show usually a CD8+, CD3+, CD2+, CD56− phenotype with expression of cytotoxic granules TIA1 and granzyme B. Rare cases of CD4+ or mixed CD4+ and CD8+ phenotype have been described usually occurring in the setting of CAEBV infection [192,196,197]. LMP1 is usually negative by immunohistochemistry. EBNA2 is always negative.

#### 5.4.3. Pathogenesis and Molecular Findings

The aetiology is unknown but its association with primary EBV infection and the racial predisposition strongly suggest a genetic defect in the host immune response to EBV [152,156,192,198,199]. The infiltrating T cells show monoclonally rearranged TCR genes. All cases harbour EBV in a clonal episomal form [152,156,196,198]. EBER *ISH* shows that the majority of the infiltrating lymphoid cells are EBV positive.

### 5.5. Aggressive NK-Cell Leukaemia

Aggressive NK-cell leukaemia (ANKL) is a rare, systemic neoplastic proliferation of NK cells, described commonly in young adults of Asian ethnicity. It was originally described as aggressive NK-cell leukaemia/lymphoma, in recognition of the variable presence of bone marrow and peripheral blood involvement [200,201]. An important feature of this disorder, which is unlike other leukaemia, is the patchy and sometimes minimal involvement of the bone marrow, even in the presence of peripheral blood disease. The WHO classification chose the name aggressive NK-cell leukaemia to allow a clear distinction between ANKL and extranodal NK/T-cell lymphoma, nasal type [202].

#### 5.5.1. Clinical Features

ANKL affects mainly young patients and the prognosis is poor. Patients present acutely with fever, pancytopenia, liver failure, and fulminant clinical course with less than 2 months overall survival. Peripheral blood shows circulating leukemic cells that goes from low to very high numbers (<5% to >80%). Serum lactate dehydrogenase (LDH) levels are markedly elevated, as is circulating Fas ligand (FASL). Hepatosplenomegaly is common sometimes accompanied by lymphadenopathy, which might be the predominant symptom. In contrast to NK/T-cell lymphoma, skin lesions are rather uncommon. The disease is often complicated by hemophagocytic syndrome, coagulopathy, sepsis and multiorgan failure [200,203,204,205,206,207]. Some cases evolve from CAEBV infection of NK cell type [150,156]. ANKL and systemic EBV+ T-cell lymphoma of childhood are similar in their clinical presentation but differ in the proliferating EBV infected cell (NK cell vs. T cell).

#### 5.5.2. Morphology

**Figure 11:**
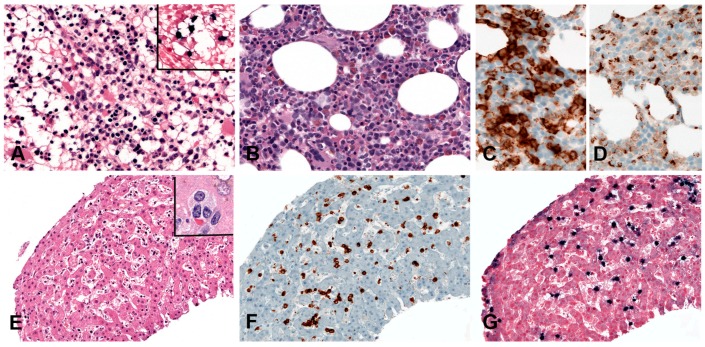
Aggressive NK-cell leukaemia. (**A**) Bone marrow biopsy with a subtle lymphoid infiltrate difficult to identify with H&E stains (H&E, 400×). Insert: EBER *in-situ* hybridization shows EBER+ cells (400×). (**B**) Hypercellular bone marrow biopsy with a clear medium-sized cell infiltrate with irregular nuclei (H&E, 400×). (**C**) The infiltrating cells are CD56 positive. (**D**) TIA1 is also positive (magnification, C, D, 400×). (**E**) Liver biopsy with dilated sinuses and a subtle infiltrate of small to medium-sized cells (H&E, 200×). Inset: The cells show atypia with irregular nuclei and one conspicuous nucleolous (H&E, 630×) (**F**) CD56 is positive in the infiltrating lymphocytes indicative of the NK-cell derivation. (**G**) EBER *in-situ* hybridization is positive (magnification, F, G, 200×).

The circulating leukemic cells exhibit a broad range of appearances, from normal looking granular lymphocytes to atypical-looking large granular lymphocytes with irregular nuclear foldings, open chromatin, prominent nucleoli and abundant pale or basophilic cytoplasm. In the bone marrow, the infiltrate is often subtle and patchy, difficult to recognize with conventional H&E stain (Figure 11A) but can also be diffuse and intermingled with increased amount of histiocytes with hemophagocytosis (Figure 11B–D). In the liver, spleen and lymph nodes the infiltrate can be massive or subtle and patchy like in the bone marrow (Figure 11E–G). The neoplastic cells have a characteristic NK cell phenotype with expression of CD2, CD3ε, CD56 and cytotoxic granules TIA1 and granzyme B (Figure 11C,D,F). Surface CD3, CD5 and CD57 are negative. In contrast to extranodal NK/T-cell lymphoma, nasal type, the cells in ANKL often express CD16 (in 75% of the cases) [207]. The neoplastic cells express FASL and FAS (CD95) with high levels found in the serum [208,209].

#### 5.5.3. Pathogenesis and Molecular Findings

The aetiology is unknown but the strong association with EBV suggests a pathogenetic role of the virus [201]. Nevertheless, in the last years, cases of EBV-negative ANKL have been reported [210,211]. EBV-negative ANKL seems to be indistinguishable clinically and pathologically from the EBV-positive cases. However, patients with EBV negative disease tend to be older at presentation (median age 63 years) [211]. Some of the EBV-negative cases have been reported to evolve from chronic lymphoproliferative disorder of NK cells [207,211,212]. A complex karyotype with recurrent chromosomal abnormalities such as gains of 1q23.1–1q24.2 and 1q31.3–q44 and losses of 7p15.1–p22.3 and 17p13.1 are characteristic of ANKL [213,214]. Malignant NK cells constitutively express FASL and the elevated FAS level detected in serum is believed to be responsible for liver necrosis and multiorgan failure [215]. Little is known about the molecular pathogenesis of ANKL. However, recent studies have identified frequent mutations leading to activation of the JAK/STAT pathway including *STAT5B* and *STAT3* gene mutations [211,216], *TP53* mutations have also been reported [210].

### 5.6. Extranodal NK/T-Cell Lymphoma, Nasal Type

Extranodal NK/T cell lymphoma, nasal type is considered the prototype of EBV-driven T/NK-cell lymphomas. It is a predominantly extranodal lymphoma of NK-cell lineage, and less often of T-cell lineage. Because of its destructive facial midline characteristics, it was referred to in the past as lethal midline granuloma. NK-cells are part of the innate immune system and reside predominantly in extra-nodal sites, where they proliferate rapidly in response to antigens without prior sensitization [217]. The normal NK-cell function and distribution might explain why its malignant counterpart presents mainly in extranodal sites and are aggressive disorders. Extranodal NK/T-cell lymphomas are characterized by angioinvasion and angiodestruction, prominent necrosis, expression of cytotoxic molecules and EBV association [218]. It is much more common in Asia and the indigenous population of Mexico, Central and South America. The genetic link between Asians and Native American populations are hypothesized to be the basis of this geographic distribution. It comprises approximately 3–10% of all lymphomas in East Asia, around 6% in Latin America but less than 1% in Western countries [219,220]. It is more common in males than in females.

#### 5.6.1. Clinical Features

The usual site of presentation is the upper aerodigestive tract including nasal cavity, nasopharynx, paranasal sinuses, and palate [218,221]. The initial complaints are usually local symptoms such as nasal obstruction, discharge and epistaxis. In more advanced stages there is an extensive destructive mid-facial lesion. The lymphoma extends to the nasopharynx, paranasal sinuses, orbit, oral cavity, palate and oropharynx. The disease may disseminate to various sites but bone marrow infiltration is uncommon [222]. Nevertheless, most patients (80%) present with localized disease (stage I–II), whereas patients presenting with extranasal localization are more frequently in advance stage at diagnosis (stage III–IV, 60%) [203]. Approximately 20% of the cases present outside the nasal region, being the skin the most frequent localization. Other preferential sites of extranodal involvement are soft tissue, gastrointestinal tract, testes and central nervous system (CNS). Secondary lymph node involvement might occur [203,223]. Skin lesions are commonly nodular, often with ulceration. Intestinal perforation and/or gastrointestinal bleeding are the common symptoms when presenting in the gastrointestinal tract. Systemic symptoms such as fever, malaise and weight loss are not rare [203,224,225]. Many patients that present with “primary” extranasal localizations have occult nasal lymphoma [220]. Therefore, careful nasal cavity inspection is recommended. The prognosis is variable, with some patients responding well to therapy and others dying of disseminated disease despite aggressive therapy.

#### 5.6.2. Morphology

**Figure 12:**
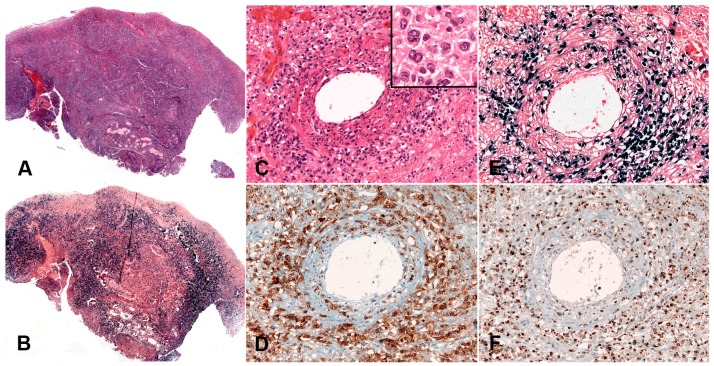
Extranodal, NK/T-cell type, nasal type. (**A**) Nasal biopsy shows a dense lymphoid infiltrate with extensive ulceration of the epithelium and destruction of adnexae and blood vessels (H&E, 25×). (**B**) Same biopsy stained with EBER *in-situ* hybridization reveals the dense EBER+ infiltrate (25×). (**C**) Medium-sized arteria with angioinvesion and angiodestruction (H&E, 200×). Inset: higher magnification shows the atypical cell infiltrate, with irregular nuclei and abundant cytoplasm (H&E, 630×). (**D**) The tumour cells are CD56 positive. (**E**) The cells are EBER positive. (**F**) TIA1 is also positive in the tumour cells (magnification, D–F, 200×).

The morphological characteristics are the same regardless of the primary infiltration site [218]. Mucosal sites show extensive ulceration with coagulative necrosis and karyorrhexis (Figure 12A,B). Angiocentricity and angiodestruction are frequently present with fibrinoid necrosis in the blood vessels. (Figure 12C–F) The cytological spectrum is rather broad. Tumour cells might be small, reactive-looking, medium-sized or large and pleomorphic. The admixed inflammatory infiltrate might mimic an inflammatory process [226]. The typical immunophenotype is CD2+, CD5−, CD56+, surface CD3− and CD3ε+ with expression of cytotoxic molecules (granzyme B, perforin and TIA1) (Figure 11C–F) [219,227]. CD56 is not specific for extranodal NK/T-cell lymphoma. There are also cases of extranodal NK/T-cell lymphoma that do not express CD56; however, expression of cytotoxic granules and EBV+ is required to make the diagnosis (Figure 12B,E,F). The clinical features and morphology of the CD56-negative cases are indistinguishable from the CD56-positive group [203]. Other T and NK-cell associated markers are usually negative (CD4, CD8, CD16 and CD57). There is a small subset of cases that have a cytotoxic T-cell phenotype with expression of CD8 and T-cell receptor either gamma-delta or alpha-beta [228]. CD30 is often expressed, as well as other activation markers such as CD25, FAS (CD95), FASL (CD178) and HLA-DR [220,229]. The FAS-FASL system has been postulated to play a role in tumour apoptosis and vascular damage [230,231].

#### 5.6.3. Pathogenesis and Molecular Findings

EBV appears to play an important aetiological role in the genesis of extranodal NK/T-cell lymphoma, nasal type. EBV exists in a clonal episomal form in the tumour cells and shows a type II latency pattern, although LMP1 cannot always be demonstrated in paraffin-embedded tissue. The EBV strain is usually type A, with a high frequency of 30 base-pair deletion of the *LMP1* gene that might reflect the geographic distribution of EBV type A strain [232,233]. Immunosuppression might play a role since it has been reported to occur in the post transplantation setting [234]. TCR genes are usually in germline configuration; however, clonal TCR rearrangements are reported to occur in 10–40% of cases, presumably the cases of cytotoxic T-cell derivation [220,228,235]. The gene expression profile analysis has demonstrated that extranodal NK/T-cell lymphomas of NK-cell and gamma delta T-cell derivation cluster together justifying to group these lymphomas as NK/T cell lymphomas irrespective of their NK- or T-cell derivation [236]. The commonest cytogenetic abnormality are del (6)(q21q25) or i(6)(p10); however, it is unclear whether these alterations represent a primary event or rather a marker of progression [237]. In the last years mutational analysis have discovered recurrent mutations and deletions in members of the JAK/STAT signalling pathway (JAK3, *STAT3* and *STAT5B*), in the gene encoding the RNA helicase *DDX3X*, in tumour suppressor genes including *TP53*, *MGA*, *PRDM1*, *FOXO3*, *HACE1* and in the transcription corepressor *BCOR*, among others [238,239,240,241] P53 protein overexpression occurs in 45–85% of the cases; however, *TP53* mutations are found in 24–62% of the cases and seem to correlate with large-cell morphology and advanced state at diagnosis [219,242].

### 5.7. Primary EBV-Positive Nodal Peripheral T- or NK-Cell Lymphoma

Primary EBV-positive nodal T- or NK-cell lymphomas have been incorporated in the revised 2016 WHO classification, not as a distinct entity, but as a provisional subgroup of PTCL, NOS [218]. These cases have a monomorphic pattern of infiltration and lack the angiodestruction and necrosis seen in extranodal NK/T-cell lymphomas.

#### 5.7.1. Clinical Features

This lymphoma presents mainly in elderly patients with a median age of 62 years. They often present with advanced stage disease (III–IV) and B symptoms. The disease has an aggressive clinical course with a median survival of 4 months. Interestingly, some reported cases seem to be associated with immunodeficiency. By definition, this is primarily a nodal disease without nasal involvement [243,244,245,246].

#### 5.7.2. Morphology

**Figure 13:**
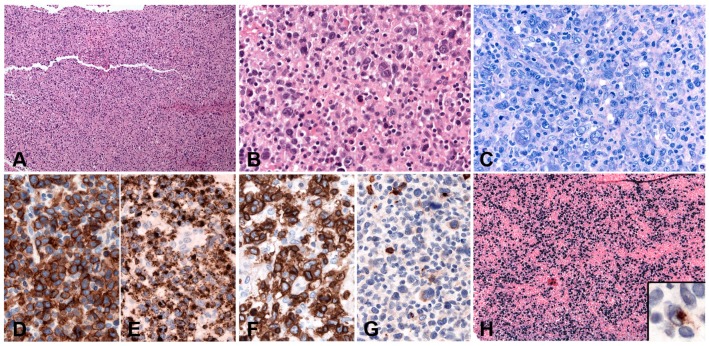
Primary EBV+ nodal T cell lymphoma. (**A**) Lymph node with complete destruction of the normal architecture by a diffuse lymphoid infiltrate (H&E, 100×). (**B**) Higher magnification shows that the infiltrate is composed of large atypical, pleomorphic cells some resembling Hodgkin and Reed-Stenberg cells (H&E, 400×). (**C**) Giemsa stain highlights the cytological features of the neoplastic cells (Giemsa, 400×). (**D**) The tumour cells are CD3 positive. (**E**) TIA1 is positive. (**F**) Note the strong, homogeneous expression of CD30. (**G**) CD56 is positive only in rare malignant cells (immunohistochemistry, D–G, 400×). (**H**) EBER is positive in the majority of tumour cells (*in-situ* hybridization, 100×). Inset: LMP1 is positive indicating an EBV latency type II (magnification, 400×).

Lymph nodes show a diffuse infiltration of pleomorphic cells with anaplastic or plasmablastic morphology (Figure 13A–C). Reed-Sternberg-like cells are often found. Some cases show necrosis and many apoptotic bodies. The immunophenotype is usually of a cytotoxic T-cell with CD3+, CD8+, TIA1+ (Figure 13D–F). Expression of CD56 is rare (Figure 13G), as well as CD4. Most of the cases have expression of TCR alpha-beta (58%), some are TCR gamma-delta positive (13%) and a third of the cases (29%) are TCR-silent [244]. The TCR silent cases are characterized by high CD30 expression (Figure 13D), an important pitfall with anaplastic large cell lymphomas (ALCL). EBER *ISH* is positive in the majority of tumour cells with expression of LMP1 consistent with an EBV latency type II (Figure 13H).

#### 5.7.3. Pathogenesis and Molecular Findings

TCR genes are usually monoclonally rearranged. A recent GEP and cytogenetic analyses revealed frequent losses of 14q11.2, which correlates with loss of TCR loci and confirms the T-cell origin. This group was characterized by upregulation of PD-L1, CD2 and CD8, and downregulation of CD56 [246]. Interestingly, PD-L1 upregulation did not correlate with EBV expression. The GEP analysis demonstrated significant enrichment of immune response genes with upregulation of genes associated with cytotoxic activation and downregulation of genes associated with T- and B-cell activation.

## 6. Conclusions

The 2016 revision of the WHO classification maintains the same principles of the 2008 edition, which is to recognize distinct entities on the basis of morphology, immunophenotype, genetic changes, and clinical features. There is a better understanding of the EBV+ LPDs that has resulted in the recognition of EBV+ MCU as a specific clinico-pathological entity. The importance of the EBV+ LPD of childhood has resulted in the addition of CAEBV infection—systemic and cutaneous forms—and changes in nomenclature that convey better the clinical features of different diseases. EBV+DLBCL is now called NOS to acknowledge that these cases can present over a wide age range, although the disease usually occurs in individuals over 50 years. Primary EBV+ nodal T- or NK-cell lymphoma has been included as a variant of PTCL, NOS, waiting for more data that confirm that it is a separate entity. The major contribution of molecular studies that has shed light onto molecular pathways has also been incorporated in this new classification.

## Figures and Tables

**Table 1:** EBV-associated B-cell lymphoproliferative disorders.

**1. B-cell lymphoproliferations EBV-associated** Infectious mononucleosis EBV+ diffuse large B-cell lymphoma, NOS EBV+ mucocutaneous ulcer Diffuse large B-cell lymphoma associated with chronic inflammation Fibrin associated diffuse large B-cell lymphoma Lymphomatoid granulomatosis
**2. B-cell lymphomas that might be EBV-associated** Plasmablastic lymphoma Burkitt lymphoma Classic Hodgkin Lymphoma
**3. B-cell lymphomas rarely EBV-associated** Chronic lymphocytic leukaemia Myeloma/Plasmacytoma
**4. Immunodeficiency-associated lymphoproliferative disorders** Lymphoproliferations associated with primary immune deficiencies Lymphomas associated with HIV Post-transplant lymphoproliferative disorders (PTLD) Non-destructive PTLD Polymorphic PTLD Monomotphic PTLD Monomorphic B-cell PTLD Monomorphic T/NK-cell PTLD Other iatrogenic immunodeficiency-associated lymphoproliferative

**Table 2:** EBV-associated T and NK-cell lymphoproliferative disorders.

**1.** **EBV-positive T-cell and NK cell lymphoproliferative diseases of childhood** Chronic Active EBV infection of T- and NK-cell type Systemic form Cutaneous form Chronic Active EBV infection of T- and NK-cell type Severe mosquito bite allergy Systemic EBV-positive T-cell lymphoma of childhood **2.** **Aggressive NK-cell leukaemia** **3.** **Extranodal NK/T-cell lymphoma, nasal type** **4.** **Primary EBV-positive nodal T- or NK-cell lymphoma**

**Table 3:** Summary of B-cell lymphomas (non-Hodgkin and classic Hodgkin) EBV-associated.

	Anatomic Sites and Distribution	EBV (%)	LMP1	EBNA2	Morphologic Features	Immunophenotypic Features and Clonality	Pathogenesis	Main Genetic Alterations
**EBV+ DLBCL, NOS**	LN or extranodal sites	100	+/−	−/+	Large cells; centroblastic or HRS-like morphology, TCRBL-like; angioinvasion and necrosis	Mainly post GC phenotype: CD45+/−, Pan-B cell markers+, CD138−, CD10−, BCL6+/−. IRF4/MUM1+; 68%, CD30+ and CD15+. IGH monoclonal.	Immunosenescence, Immune evasion (PDL2 upregulated)	NFkB and JAK/STAT pathways activated. GEP: “host immune response”
**EBV+ mucocutaneous ulcer**	Oral cavity, skin and GI tract	100	+	−/+	Circumscribed ulcer with large cells immunoblastic or Hodgkin-like features	Post GC phenotype: CD45+, Pan-B cell markers+, CD138−. CD10−; variable positivity for BCL6, IRF4/MUM1+. ~50% co-express CD30 and CD15. IGH monoclonal	Immuno-senescence or immune-suppression. Postulated immune sequestration	Not known common T-cell oligoclonality or monoclonality
**DLBCL-associated with chronic inflammation**	Pleural cavity, cysts and other confined spaces	100	+	+	Morphology similar to conventional DLBCL	Post GC phenotype: Pan-B cell markers+, CD10−, BCL6+/−, CD30+, MUM1/IRF4+; plasmablastic phenotype possible (pan-Bcell markers−, CD138/MUM1+). IGH monoclonal	Immune sequestration in confined spaces due to prolonged inflammation	High genetic complexity; common *TP53* mutation, *MYC* amplification and *TNFAIP3* deletion
**Fibrin-associated DLBCL**	Cardiac myxoma, cardiac fibrin thrombi, implants	100	+	+	Large cells centroblastic, immunoblastic or plasmablastic features	Post GC phenotype: CD45+, CD20+, PAX5+, CD79a+, BCL6+/−, MUM1/IRF4+, CD30+, MYC (<50%), p53 (<30%). IGH monoclonal	Immune sequestration in avascular fibrin masses	Low complexity of genetic abnormalities
**Lymphomatoid granulomatosis**	Lung, CNS, skin, liver or kidney	100	−/+	−/+	Large cells with centroblastic, immunoblastic or HRS-like features in a T-cell reactive background; Angioinvasion and necrosis	Post GC phenotype: CD45+, Pan-B cell markers+, CD30+, CD15−; IGH monoclonal.	Underlying inherent immunosuppression	Alterations of oncogenes not detected
**Plasmablastic lymphoma**	Solid extranodal masses, GI tract, LN	70–80	−/+	−	Plasmablastic, immunoblasticor anaplastic	Terminally differentiated B-cell: CD45−, CD20−, PAX5−, CD79a−/+, CD138+, CD38+, CD10−/+, CD56−/+, BCL6−, MUM1/IRF4+, BLIMP1+, XBP1+, cIgG; IGH monoclonal	EBV driven B-cell proliferation in an immunosuppressed setting	Complex karyotypes; *MYC* rearrangement (>50%); *PRDM1* mutations (49%)
**Burkitt lymphoma -Endemic -Sporadic -HIV+**	LN or extranodal sites	100 5–80 30–40	−	−	monotonous medium-sized blasts without prominent nucleoli “Starry sky” appearance;	GC phenotype: CD45+, Pan-B cell markers+, CD10+, BCL6+, BCL2−, sIgM+, Ki67 100%, MYC 100% IGH monoclonal	Synergistic effect of EBV and *MYC*; EBV may not be essential.	*MYC*/IG translocation (>90%); *TP53* (30%), *TCF3* (70% sBL) mutations.
**Classic Hodgkin lymphoma**	LN	20–100	+	−	HRS cells in a typical inflammatory background	CD45−, CD20−/+, CD79a−/+, PAX5+ (weak), OCT2−, BOB1−, Ig−, CD30+, CD15+, CD10−, BCL6−/+, MUM1+	EBV pathogenetic role likely in some cases “Crippling” mutations of the IGH genes. Aberrant Ig transcription	NFkB and JAK/STAT pathways activated. GEP: “Host immune response” Altered PD1-PD-L1 signalling

DLBCL: diffuse large B-cell lymphoma; NOS, not otherwise specified: CB: centroblastic cytology; IBL: immunoblastic cytology; IGH: Immunoglobulin heavy chain gene; EBV: Epstein-Bar virus; LMP1: Latent membrane protein 1; EBNA2: EBV-encoded nuclear antigen 2; LN: Lymph nodes; CNS: central nervous system; GI: gastrointestinal; BM: bone marrow; Ig: Immunoglobulin; GEP: Gene expression profiling signature. sBL: sporadic Burkitt lymphoma; HRS: Hodgkin-Reed-Sternberg.

**Table 4:** Summary of the EVB-associated T- and NK-cell lymphoproliferative disorders.

	Anatomic Sites and Distribution	EBV (%)	LMP1	Cell of Origin	Morphologic Features	Immunophenotypic Features and Clonality	Pathogenesis and Main Genetic Alterations
**CAEBV, systemic form**	spleen, liver, LN, extranodal sites	100	+/−	T cell (59%), NK cell (41%)	There are no changes suggestive of a malignant LPD	CD4 > CD8 CD56+ (41%) TCR monoclonal in 84%	Unknown. Racial predisposition, defective EBV immune response
**Hydroa vacciniforme-like LPD**	skin	100	+/−	T cell (85%) αβ and γδ rarely NK cell (15%)	Intraepidermal spongiotic vesicles. Periadnexal and perivascular infiltrate with angiodestruction	CD8 > CD4 CD56+ (15%) CD30 often express TCR often monoclonal	Unknown. Racial predisposition, defective EBV immune response
**Severe mosquito bite allergy**	skin	100	−	NK cell	Small reactive looking lymphocytes to large atypical neoplastic cells	CD3ε+, CD56+, TIA1+, granzyme B+, CD30+ TCR polyclonal	CD4+ T cells response to mosquito salivary gland secretion that reactivates EBV infection
**Systemic EBV+ T-cell lymphoma of childhood**	Spleen, liver, LN	100	+/−	T cell (CD8 > CD4)	Hemophagocytosis with T cell infiltrates in LN, liver, spleen	CD8+, CD3+, CD2+, CD56−; rare cases CD4+ TCR monoclonal	Unknown. Racial predisposition, defective EBV immune response
**Aggressive NK-cell leukaemia**	BM, PB, spleen, liver	95	−	NK cell	Hemophagocytosis. PB and BM infiltrated with atypical granular lymphocytes with broad cytological spectrum	CD3ε+, CD56+, CD2+, TIA1+, granzyme B+, CD16+ (75%), CD95+ (FAS), FASL+, CD3−, CD5, CD57−. TCR polyclonal	Unknown. Racial predisposition, defective EBV response. Complex karyotype with gains of 1q23.1–1q24.2 and 1q31.3–q44 and losses of 7p15.1–p22.3 and 17p13.1. *STAT3*, *STAT5B* and *TP53* mutations
**Extranodal NK/T-cell lymphoma, nasal type**	Nasal cavity, nasopharynx, palate, skin, GI, testis, CNS	100	+/−	NK cell rarely T cell	Ulceration, coagulative necrosis, karyorrhexis, angiocentricity and angioinvasion. Cytological spectrum broad	CD3ε+, CD2+, CD56+, TIA1+, granzyme B+, CD30+, CD3−, CD5−, CD16−, CD57−, CD4/CD8−. TCR polyclonal	Racial predisposition, defective EBV immune response. Cytogenetic alterations with del (6)(q21q25) or i(6)(p10). Recurrent mutations in *STAT3*, *STAT5B*, *DDX3X*, *TP53*, *MGA*, *PRDM1, FOXO3*, *HACE1*, *BCOR*
**Primary EBV+ nodal T- or NK-cell lymphoma**	LN	100	+/−	T cell TCRαβ (58%), TCRγδ (15%), TCR silent (29%), rarely NK cell	LN with diffuse infiltration of pleomorphic, anaplastic or HRS-like cells	cytotoxic T-cell phenotype: CD3+, CD8+, TIA1+, granzyme B+, CD56−, CD4−, CD30+/−. TCR monoclonal	Racial predisposition, defective EBV immune response. Cytogenetic alterations with frequent losses of 14q11.2. GEP: enrichment of immune response genes and genes associated with cytotoxic activation. PD-L1 upregulation

LPD: lymphoproliferative disorder; NOS, not otherwise specified: NK: natural killer; PB: peripheral blood; HRS-like: Hodgkin and Reed-Sternberg-like; GEP: Gene expression profile; EBV: Epstein-Bar virus; LMP1: Latent membrane protein 1; LN: Lymph nodes; CNS: central nervous system; GI: gastrointestinal; BM: bone marrow; CAEBV: Chronic active EBV infection.
